# Comparing watershed-based spatial units and administrative boundaries for malaria prediction in the Peruvian Amazon

**DOI:** 10.1371/journal.pgph.0007553

**Published:** 2026-11-02

**Authors:** Antony Barja, Edson J. Ascencio, Bryan Fernandez-Camacho, Luis C. Revilla, Jazmin Qquellon, Gabriel Carrasco-Escobar

**Affiliations:** 1 Health Innovation Laboratory, Institute of Tropical Medicine “Alexander von Humboldt”, Universidad Peruana Cayetano Heredia, Lima, Peru; 2 Yale School of Public Health, New Haven, Connecticut, United States of America; University of Cape Town, SOUTH AFRICA

## Abstract

Malaria continues to pose a major public health threat in the Peruvian Amazon, where climate variability influences transmission patterns. This study assessed whether watershed-based spatial units (hydrobasins) outperform traditional administrative boundaries in predicting *P. vivax* and *P. falciparum* cases in Loreto, Peru. A retrospective ecological analysis was conducted using village-level geocoded malaria case data from 2009 to 2018, aggregated at the district level and three hydrobasin levels (levels 6, 7, and ANA). Environmental and meteorological variables were derived from satellite data and incorporated into Zero-Inflated Negative Binomial regression and machine learning models (Random Forest, Support Vector Machine, and XGBoost). Model performance was evaluated using Root Mean Squared Error (RMSE) and Akaike Information Criteria (AIC). Among 297,324 malaria cases analyzed (79.48% *P. vivax*, 20.52% *P. falciparum*), hydrobasin level 7 consistently showed superior predictive accuracy. For *P. falciparum*, hydrobasin level 7 consistently produced the lowest prediction errors across modelling approaches. Among machine learning models, XGBoost achieved an RMSE of 15.79 (95% CI: 10.42-21.03), whereas the corresponding ZINB-PCA model achieved an RMSE of 39.19 (95% CI: 17.09-62.24). District- and ANA-based ZINB-PCA models yielded RMSE values of 50.89 (95% CI: 23.65-77.87) and 59.22 (95% CI: 33.26-82.21), respectively. For *P. vivax*, XGBoost achieved an RMSE of 38.44 (95% CI: 26.90-49.19), whereas the corresponding ZINB-PCA model achieved an RMSE of 63.87 (95% CI: 46.38-80.95). District-, hydrobasin level 6-, and ANA-based ZINB-PCA models produced RMSE values of 102.81 (95% CI: 73.15-131.77), 175.87 (95% CI: 116.08-232.61), and 263.78 (95% CI: 144.98-389.87), respectively. These findings suggest that the choice of spatial unit affects predictive accuracy, with hydrobasin level 7 producing the lowest prediction errors; the associations captured by the models are predictive and are not interpreted as causal effects. Integrating hydrologically defined boundaries into surveillance systems may enhance malaria control strategies in environmentally complex regions.

## 1 Introduction

Malaria remains concentrated in the Amazon. In the Region of the Americas the World Malaria Report estimated 536,670 malaria cases during 2024, and although the region reduced its burden by 58% between 2010 and 2014, reported cases have risen again since 2015 [1]. Transmission is focal and clusters in a small number of ecoregions, of which the Amazon is the most important [2], and it is dominated by *Plasmodium vivax* transmitted by the *Nyssorhynchus* (*Anopheles) darlingi* mosquito [3]. In Peru, despite the control and prevention activities carried out by the government during the last decades, malaria cases caused by *P. vivax* and *P. falciparum* remain a major public health priority [4]. By 2024, Loreto department accounts for more than 95% of Peruvian malaria cases (82% of cases due to *P. vivax* and 18% of them by *P. falciparum*) according to the National Center for Epidemiology, Prevention and Diseases Control of the Ministry of Health of Peru (MoH) [5].

Climate variability and seasonality are related to malaria occurrence and its spatio-temporal distribution [6,7]. Several studies highlighted the malaria parasite and its mosquito vector sensitivity to variations in temperature, precipitation, evapotranspiration, soil moisture, and vegetation variables [8–10]. In addition, the synergistic action of rainfall, temperature, and humidity influence the duration of larval development, the parasite extrinsic incubation period (EIP), and the vector longevity, competence, fecundity, and biting rate [11–14]. Runoff and evapotranspiration are closely associated with environmental conditions suitable for mosquito reproduction and breeding, providing temporary water pools in topographic depressions parallel to the surface [15,16]. Finally, vegetation indexes such as the Normalized Difference Vegetation Index (NDVI), the Enhanced Vegetation Index (EVI), and the Soil Adjusted Vegetation Index (SAVI) measure the vegetation dynamics, abundance, and its spatial distribution; which are closely related to vector behavior and habitat suitability by providing a resting place for adult mosquitoes [17,18]. All these meteorological and environmental variables showed a high heterogeneity that influences the transmission of malaria parasites [19].

Malaria lifecycle and its distribution are closely related to environmental factors, and the assessment of this relationship usually considers data aggregated at governmental administrative boundaries such as districts, provinces, or municipalities, depending on the study area and design [20]. Spatial and epidemiological analyses that use these administrative boundaries are useful to understand malaria transmission dynamics and hotspots localization to target, tailor, and intensify cost-effective malaria intervention programs [21]. However, in most cases, administrative boundaries follow arbitrary and historical divisions that are incapable of reflecting micro-environmental dynamics. Conversely, ecological boundaries such as watersheds or hydrobasins are characterized by the interaction of consistent climatic variables [22,23]. These natural boundaries are shaped by terrestrial and aquatic ecosystem topographies instead of political boundaries and are controlled by specific spatio-temporal land-surface interactions of hydrological and biogeochemical processes [24]. Commonly, these natural formations are important sources of mosquito breeding sites and are considered natural barriers to hydrological processes [25]. In addition, hydrobasins represent the main pathways for outdoor activities in the Amazon Region by involving human mobility between villages, which is a key condition for high malaria transmission levels in rural areas [26]. These boundaries characterize watershed homogeneous zones according to the type and use of soil, vegetation cover, and climate properties [27]. The usefulness of watershed-based boundaries has been observed in the analysis of meteorological variability phenomena between different areas sharing common characteristics [28].

Efforts to anticipate malaria have produced several families of forecasting frameworks, including climate-driven statistical models extended with seasonal climate forecasts [29], mechanistic models that simulate vector and parasite dynamics [30], Bayesian spatiotemporal models [31], and machine learning approaches applied to malaria risk in the region [10]. Most of these models are fitted to administrative units. A scoping review of malaria forecasting in the region found that all studies included used political administrative units [32], and another review of thirty early warning systems across sixteen countries reports the same practice [33]. The reporting standard for this field contains nineteen items, none of which asks authors to state or justify the spatial scale of analysis [34]. This matters because the modifiable areal unit problem (MAUP), which is a statistical bias in spatial analysis, could be affected by the scale effect, in which results change as data are aggregated into fewer and larger units, and a zoning effect, in which results change across alternative partitions holding a fixed number of units [35]. The severity of these effects depends on how homogeneous units are with respect to the drivers.

The relevance of the MAUP to malaria follows from the spatial scale at which transmission is generated. For example, larval habitats of *Nyssorhynchus darlingi* have been shown to form through the flooding and drainage of rivers, streams and topographic depressions [15,16,25]. Human exposure in the Amazon is also organised along river networks, where settlements, outdoor activities and movement between villages follow the drainage [26]. Additionally, when cases and environmental covariates are averaged over units that combine contrasting habitats, the unit-level covariate may no longer describe the conditions under which infection occurred. The exposure-response relationship estimated from aggregated data may then differ from the relationship operating at the scale of transmission, which is the aggregation bias that underlies the MAUP [35]. Watershed-based boundaries are delimited by drainage divides. Runoff, flooding and soil moisture, the processes that create and remove breeding habitat, are therefore expected to be shared within a basin to a greater extent than within units drawn for administrative purposes [22–24,27]. Units that are more homogeneous with respect to these drivers would be expected to reduce this mismatch and could change the accuracy of predictions derived from environmental covariates.

Therefore, it is hypothesized that watershed-based boundary units or hydrobasins, which are internally coherent with respect to the environmental processes generating transmission, characterise the climate-malaria relationship better than administrative units drawn for political and logistical reasons. To better inform surveillance and modelling strategies, we aimed to compare the predictive performance of watershed-based and administrative boundaries for estimating *P. vivax* and *P. falciparum* malaria cases in the Peruvian Amazon.

## 2 Methods

### 2.1 Ethics statement

This study used anonymized secondary data obtained from the routine malaria surveillance system of the Peruvian Ministry of Health (MoH). The dataset did not contain any personally identifiable information, and all analyses were conducted at aggregated spatial levels. According to national regulations and institutional guidelines, studies based on secondary anonymized surveillance data do not require formal ethical approval. Therefore, no ethics committee approval was necessary for this study.

### 2.2 Study design

Retrospective ecological study that used secondary panel data of monthly village-level geocoded malaria cases aggregated at the areal level based on administrative units and three hydrobasins levels in the Loreto Region, Peru: hydrobasin level 6, hydrobasin level 7, and the watersheds delimited by the National Water Authority (ANA by its Spanish acronym). Multiple meteorological variables provided by the satellite imagery datasets were collected at each areal aggregation level to model the monthly climate-malaria relationship from 2009 to 2018. This study was conducted and reported following the EPIFORGE 2020 [34] guidelines for epidemiological forecasting research.

### 2.3 Study area and population

Loreto is the northeast Peruvian region and has a total area of 368,851 km^2^, with an average population of 883,510 inhabitants (3.1% of the total national population). Loreto is divided into 53 districts, the administrative units used in this study. The predominant natural cover is characterized by amazon rainforest, with dense vegetation, low hills, and slightly undulating surfaces crossed by several large rivers (e.g., Marañon, Ucayali, Huallaga, Napo, Putumayo, and Yavarí), which confluence in the Amazon River basin [36]. The climate is predominantly rainy, warm, and humid throughout the year [37]. The maximum temperature reaches 36°C (between December and March), and the minimum is 17°C (between June and July).

### 2.4 Data sources

#### 2.4.1 Malaria cases data.

The epidemiological surveillance conducted by the MoH’s General Directorate of Epidemiology, through the NOTI platform, collected all weekly reported malaria cases for both parasite species at the village, district, and province levels [38]. We processed monthly malaria cases from 2009 to 2018 in the 1829 Loreto villages (Fig 1).

**Fig 1:**
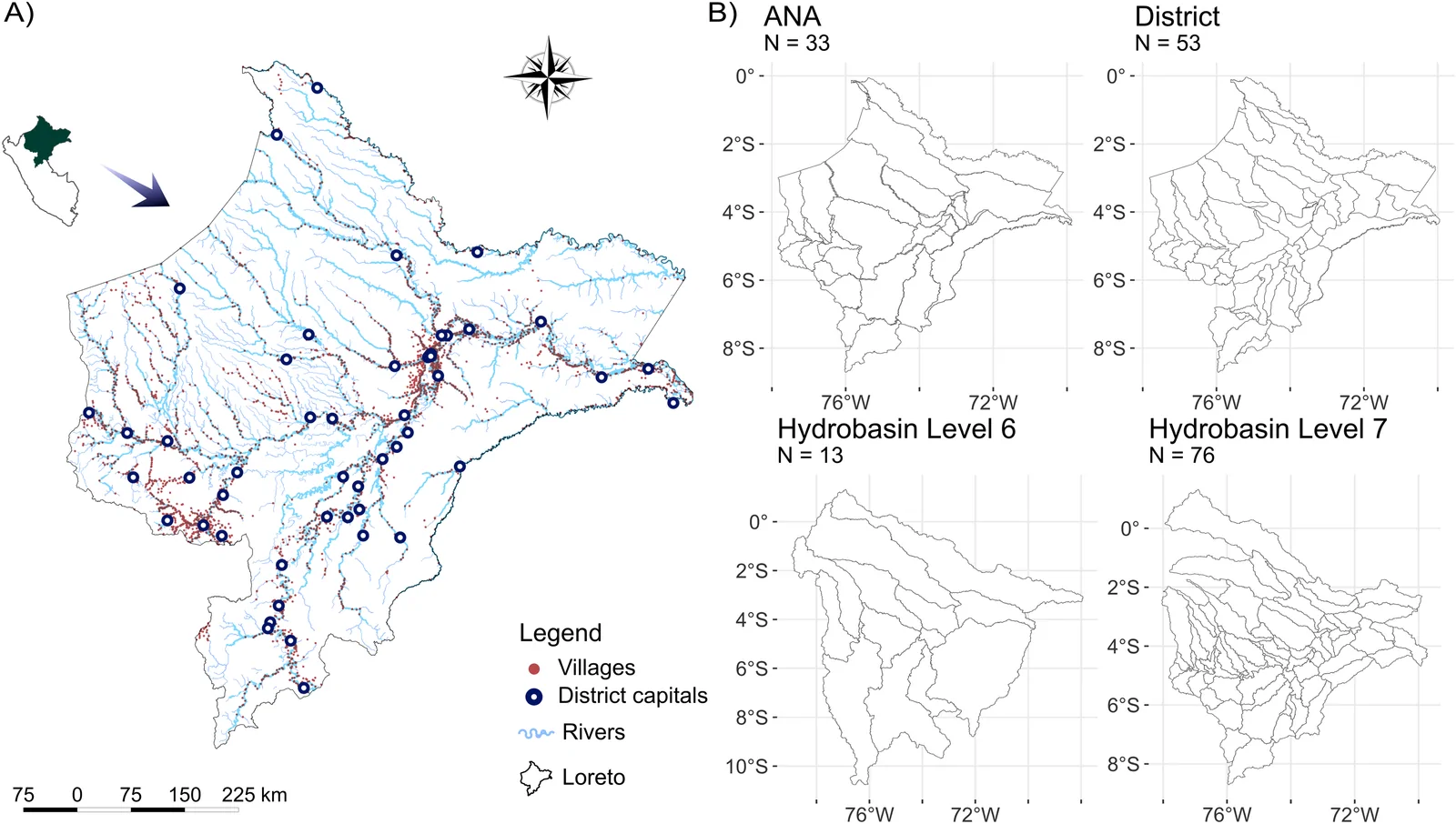
Study area in the Loreto Region, located in the north of the Peruvian Amazon. **(A)** Location map and **(B)** Watershed area units (hydro-basin and National Water Authority) and administrative (districts) boundaries included in the analysis. The map was generated with QGIS software v3.28.3 (QGIS Development Team, 2016. QGIS Geographic Information System. Open Source Geospatial Foundation Project. http://www.qgis.org/). Maps were based on public geographic data extracted from INEI, Peru contributors (https://estadist.inei.gob.pe/map) under Open Data Commons Open Database License (ODbL) 1.0 (http://openstreetmap.org/copyright).

Most of the malaria cases reported in Loreto were from those who voluntarily seek diagnosis and treatment at a healthcare facility (passive case detection). Diagnosis was based on the microscopic examination of a thick and thin capillary blood smear to identify the presence of any *Plasmodium* species in the red blood cells and their count [39].

#### 2.4.2 Pre-processing spatial data.

Malaria cases in the Loreto region were summarized from 2009 to 2018 at a monthly level (time resolution) and village level (spatial resolution). All reported cases were compiled at the community level, with each case assigned to the centroid of the respective community as point-type geometries. The spatial representation was based on a geodetic system World Geodetic System 1984 (WGS84) projection, where latitude and longitude were in decimal degrees. Data was processed using R version 4.2.1 software (R Core [Te]am 2021).This initial dataset was processed and aggregated by the boundaries considered in the study area (i.e., district level, hydrobasin level 6, hydrobasin level 7, and hydrobasin ANA) (Fig 1).

#### 2.4.3 Boundary data.

HydroSHEDS is a mapping product that provides regional and global scale hydrographic information on river networks, catchment boundaries, drainage directions, and flow accumulations. Its dataset is the result of the use of Shuttle Radar Topography Mission (SRTM) elevation imagery and the Pfafstetter coding system methodology, both of which are available at all spatial-scale levels on its official website. Levels 6 and 7 were selected a priori, before any model was fitted, on descriptive criteria: the number of units that contained villages, the number of villages per unit and the proportion of unit-months without reported cases. The selection was not based on a comparison of predictive performance across levels, because choosing the level that minimised prediction error would have made the spatial resolution a tuned parameter of the comparison under evaluation. These two levels allowed a coarse-to-fine comparison while maintaining a balanced distribution of villages and malaria cases across spatial units. Coarser levels would have produced a small number of highly aggregated units, potentially masking within-basin heterogeneity, while finer levels would have generated numerous sparsely populated units with few or no malaria cases. When the same criteria were applied to the other Hydrobasins levels, the 1,829 villages fell into only 3 units at level 5. At level 8 they fell into 240 units, 56.7% of which contained fewer than five villages, and 77.6% of unit-months had no *P. falciparum* case (S1 Table). Therefore, our study considered level 6 with 13 units, and level 7 with 76 units. These units are the Hydrobasins polygons that contained at least one village, after two pairs of adjacent level-6 polygons and three pairs of adjacent level-7 polygons that each contained villages were merged into single units

In Peru, the National Water Authority (ANA by its Spanish acronym) provides a division, codification, and systematization of hydrobasins using two international standard methodologies, Pfafstetter and cartographic method for the hydrobasins delimitation [40]. ANA-based watersheds are created using a Digital Elevation Model such as NASA’s SRTM of 30 meters spatial resolution. The final product is a map of a scale of 1:100,000 cm. ANA watershed was additionally included because it represents the official hydrological delineation used by Peru’s National Water Authority, thereby providing a standardized spatial framework for comparison.

Districts were used as the administrative spatial unit for aggregation and comparison. The district is the smallest officially recognised administrative subdivision in Peru and is nested within provinces and departments. The 53 official districts of Loreto were used [41].

#### 2.4.4 Meteorological and environmental data.

All meteorological and environmental variables analyzed in this study were derived from satellite images. For each study boundary unit inside the region, areal statistics functions were used to summarize them. All variables were processed in the R version 4.2.1 software (R Core Team 2021) [42].

Meteorological variables such as precipitation, runoff, soil moisture, and temperature were extracted from TerraClimate [43] dataset at 1 km of spatial resolution on a monthly basis from 2009 to 2018. In addition, potential evapotranspiration was extracted from the MODIS sensor (MOD16A2) [44] at 500-meter spatial resolution on a monthly basis over the same time frame. Finally, vegetation indexes (environmental variables) such as NDVI, SAVI, and EVI were derived from the National Oceanic and Atmospheric Administration-Advanced Very High-Resolution Radiometer (NOAA-AVHRR) MODIS (MOD13A1) [45] sensor data and extracted at a spatial resolution of 500 meters on a monthly basis in the same time frame (S2 Table).

### 2.5 Statistical analysis

The analysis used complementary statistical and machine learning approaches for malaria prediction. Zero-Inflated Negative Binomial (ZINB) regression was used as a likelihood-based statistical benchmark for prediction, while Random Forest (RF), Support Vector Machine (SVM), and XGBoost were used as machine learning approaches. All models were evaluated across the same spatial aggregation schemes (district, hydrobasin level 6, hydrobasin level 7, and ANA hydrobasin) to compare predictive accuracy and cross-boundary generalizability. The ZINB regression was not used for inferential estimation of environmental effects. Its coefficients can be read only as conditional associations between environmental covariates and malaria counts at the level of the aggregated spatial unit. The predictors were selected by data-driven procedures, and the design is ecological and observational. The coefficients are therefore not interpreted as causal effects or as individual-level risk factors, and no inference is drawn from their statistical significance.

#### 2.5.1 Predictors selection.

Different predictor-processing and selection strategies were used for the statistical and machine learning approaches. Two strategies were employed for predictors selection: a backward and Elastic Net [46] method for the ZINB regression and a Principal Component Analysis (PCA) for the machine learning approach.

An optimized set of predictors (variables) was identified through backward selection and Elastic Net regularization, which effectively combines L1 (LASSO) and L2 (Ridge) penalties to improve model performance and handle multicollinearity. Elastic Net was implemented with 25 lambda values to ensure optimal shrinkage, and the BIC criterion was used to identify the best-fitting model. This approach was applied for each geographic boundary (hydrobasin level 6, hydrobasin level 7, ANA hydrobasin, and districts) and for each malaria species. The final set of predictors was determined by selecting those that were consistently retained by both the Elastic Net and backward selection techniques. Further details on the composition of each predictors set are provided in S3 Table.

A PCA strategy was implemented to identify relevant patterns among variables (S3 Table). grouped into climatic, vegetation, and hydrological factors. Components were selected based on the Kaiser criterion, retaining those collectively explaining more than 80% of the variance [47]. The selected principal components served as predictors (inputs) for modelling.

#### 2.5.2 Modelling.

A Zero-Inflated Negative Binomial regression model [48] with a negative binomial family was employed to analyze malaria cases by species, addressing the presence of overdispersion. The zero-inflated negative binomial distribution was chosen on explicit comparative diagnostics rather than on the proportion of observed zeros alone. For each boundary and species, Poisson, negative binomial (NB), zero-inflated Poisson (ZIP) and ZINB models were fitted with the predictor set selected for that boundary and the same population offset. The zero component of the zero-inflated models used the same predictors as the count component. The candidate distributions were compared by the Pearson dispersion statistic of the Poisson model and by the number of zero counts expected under each fitted model against the observed number. Akaike Information Criterion (AIC) and the Bayesian information criterion (BIC) were compared within each boundary and species, the non-nested NB and ZINB models were compared with the Vuong test [49], and zero inflation relative to the NB model was tested by simulation on its randomised quantile residuals (S4 Table) [50]. A log population offset was incorporated to account for population size, ensuring that incidence rates were modeled instead of raw case counts. Community-level population estimates were obtained from the National Institute of Statistics and Informatics (INEI in Spanish). Population varied across different years and communities. For hydrobasin units, population was allocated by assigning community points to the corresponding hydrobasin polygon and aggregating population within each spatial unit. Equation 1 shows an example of the formulas used to model malaria as a function of the predictors found by variable selection for *P. falciparum* in set 3 (See S3 Table):


$$\begin{array}{c} log(\mu )=\beta_{0}+\beta_{1}(vs)+\beta_{2}(ro)+\beta_{3}(so)+\beta_{4}(le)+\beta_{5}(ev)+ln(pob). \end{array}$$ (1)

Where μ is the mean count for the *non-zero-inflated* part and ln(pob) is included as an offset, ensuring we model the rate per population. Subsequently, cross-boundary model transfer was designed so the final models developed for each boundary were applied exclusively to the same species across different geographic boundaries. For instance, the model derived from the hydrobasin level 7 dataset for *P. vivax* (S3 Table) was applied to the datasets of the other hydrobasins but only for *P. vivax*. Two models were constructed for each geographic boundary (for *P. vivax* and *P. falciparum*), resulting in four models per malaria species. These four models were then applied across the four geographic boundaries, leading to 16 applications per species. As this process was carried out separately for both *P. vivax* and *P. falciparum*, the total number of final model applications amounted to 32.

The machine learning modelling process included three algorithms focused on a regression approach. Additionally, the population variable, along with the corresponding year and month for each analyzed spatial structure, was incorporated. The models employed were Random Forest (RF), Support Vector Machine (SVM), and XGBoost. The RF algorithm, a supervised method, was configured globally with a tuneLength of 3, which determines how many candidate values will be evaluated for each hyperparameter during the tuning process. This choice was made to balance the exploration of the hyperparameter space with the available computational efficiency. In the same context, the SVM was set up using a radial kernel, meaning the model transforms the data into a higher-dimensional feature space to capture non-linear relationships and facilitate the identification of more complex decision boundaries. XGBoost was utilized as a supervised learning algorithm that builds decision trees sequentially by optimizing a loss function. It employs Lasso (L1) regularization to promote sparse solutions and Ridge (L2) regularization to reduce the magnitude of coefficients. Each model was trained and tested using an 80/20 data split, with 80% of the data allocated for training and 20% for testing. Five-fold cross-validation was applied to enhance model generalizability and ensure robust performance. Furthermore, these models were independently implemented for each geographic boundary and malaria species. The ZINB regression model was used with the components derived from the PCA to better compare performance metrics between the algorithms.

#### 2.5.3 Hyperparameter optimisation.

Hyperparameter optimisation was conducted independently for each spatial unit and disease outcome via random search with 5-fold cross-validation, using the Root Mean Squared Error (RMSE) as the selection criterion. For RF, the number of variables randomly sampled at each split (mtry) was explored across 10 candidate values drawn at random from the range [1, p], where p denotes the total number of predictors per spatial unit. The number of trees was fixed at ntree = 500, a value widely established as sufficient for error stabilisation in ensemble models [51,52]. Optimal mtry values ranged from 4 to 14 across units and diseases (S5 Table). For XGBoost, a random subsample of 20 combinations was drawn from a full factorial grid spanning: learning rate η ∈ {0.01, 0.05, 0.10, 0.20, 0.30}, maximum tree depth d ∈ {3, 4, 6, 8}, and number of boosting rounds T ∈ {50, 100, 200, 300}. Remaining parameters were held constant (γ = 0, colsample_bytree = 1, min_child_weight = 1, subsample = 1). Optimal configurations were dominated by higher learning rates (η = 0.2-0.3) and deeper trees (d = 6–8) in most units, with the exception of hydrobasin ANA for *P. falciparum* (η = 0.1, d = 3). For SVM with RBF kernel, the penalty parameter C and kernel bandwidth σ were explored on a log_2_ scale following Hsu, Chang & Lin (2003): C ∈ {2^-3^, 2^-2^, …, 2^7^} and σ ∈ {2^-7^, 2^-6^, …, 2^1^}, from which 20 combinations were randomly sampled. Optimal C values showed a clear pattern: low penalisation (C = 1) for districts and hydrobasin ANA for *P. falciparum*, and high penalisation (C = 64) for *P. vivax* across all hydrobasin units. The same sampled candidate combinations were evaluated for every spatial unit and species, and tuning was performed within the training partition. All other hyperparameters were kept at their package defaults, and the search ranges and selected values are reported in S5 Table

#### 2.5.4 Performance metrics.

The performance of statistical and machine learning models were evaluated using the prediction error values [53] based on the RMSE and the AIC. The RMSE is a general error metric that measures the average magnitude of prediction errors. It is computed as the square root of the mean squared differences between actual values ($y_{i}$) and predicted values ($\hat{y_{i}}$), with n representing the number of data points, as shown in Equation 2. By contrast, AIC is an information-theoretic criterion that assesses model quality by balancing goodness of fit against model complexity, as shown in Equation 3. In AIC, k is the number of estimated parameters in the model, and L is the maximized likelihood of the model. Smaller RMSE values indicate better predictive accuracy, while smaller AIC values indicate a better trade-off between fit and parsimony among candidate models. Accordingly, RMSE was used to evaluate out-of-sample predictive performance. Because AIC is likelihood-based and depends on the response vector and sample size, it was not used to rank models across different spatial aggregations. AIC was interpreted only within comparable statistical models and data settings, whereas cross-boundary comparisons were based on out-of-sample predictive metrics.


$$\begin{array}{c} RMSE\;=\;\sqrt{\frac{1}{n}\sum_{i\;=\;1}^{n}(y_{i}-\hat{y_{i}}{)}^{2}} \end{array}$$ (2)

$$\begin{array}{c} AIC\;=2k-2ln(L)\; \end{array}$$ (3)

To quantify the uncertainty of predictive performance, 95% confidence intervals (95% CIs) for the RMSE were estimated using a cluster bootstrap procedure with 5,000 resamples. Resampling was performed at the geographic-unit level since repeated monthly observations within the same geographic unit were not statistically independent. In each bootstrap replicate, spatial units (districts or hydrobasins) were sampled with replacement, all observations belonging to the selected units were retained, and the RMSE was recalculated using the fixed model predictions. The 95% CIs were obtained using the percentile method (2.5th and 97.5th percentiles of the bootstrap distribution). The same bootstrap procedure was applied to both the statistical and machine learning models to ensure a consistent estimation of predictive uncertainty across modelling frameworks. These intervals describe the uncertainty of the RMSE, a summary statistic of model performance, under resampling of spatial units. They are not prediction intervals and do not quantify the uncertainty of the predicted monthly case count for an individual spatial unit or month.

Pairwise differences in predictive accuracy were further evaluated using Diebold-Mariano tests [54]. Each test compared two models on the same evaluation boundary, so that both models produced predictions for the same spatial units and months. The competing models on an evaluation boundary were the three machine learning algorithms fitted on that boundary and the four ZINB models. Each ZINB model carried the predictor set derived in one of the four boundary systems and was fitted on the data of the evaluation boundary, following the cross-boundary model transfer described above. For month t, the loss differential was the difference between the two models in the mean squared error across the N units of the evaluation boundary. It was computed as $d_{t}=\frac{1}{N}\sum_{i=1}^{N}(e_{A,it}^{2}-e_{B,it}^{2})$, where $e_{A,it}$ and $e_{B,it}$ are the observed minus the predicted counts of models A and B for unit i in month t. This construction yielded one series of T=120 monthly loss differentials per comparison, defined on identical units for both models. Differences in the number and configuration of units between boundary systems therefore did not enter the test. Diebold-Mariano tests were not used to compare boundary systems evaluated on different units; those comparisons rest on the RMSE, its bootstrap confidence intervals and the common temporal holdout. The variance of the mean loss differential $d^{-}$ was estimated with the Newey-West heteroskedasticity- and autocorrelation-consistent estimator [55], with a truncation lag of four months. This lag was set by the automatic rule of Newey and West, $\lfloor 4(T/100{)}^{2/9}\rfloor$, which equals four for T=120 [56], and it was fixed before the tests were computed. The Harvey-Leybourne-Newbold small-sample correction was applied, with p-values from the Student t distribution on T−1 degrees of freedom [57]. Negative mean loss differences indicated lower prediction error for the first model in each comparison. Two-sided p-values below 0.05 were considered statistically significant.

#### 2.5.5 Spatiotemporal cross-validation.

Model performance was also evaluated using blocked spatiotemporal cross-validation. The data were first divided temporally, with observations through 2014 used for training and data from 2015 to 2018 reserved for testing. Within the training period, spatial folds were defined according to the geographic unit of analysis (districts or hydrobasins). Entire spatial units were left out in each fold, so model performance was evaluated in locations not included during training. Predictions were then generated for the corresponding held-out spatial units during the 2015–2018 test period. This provided a direct assessment of performance when both the location and time period differed from those used for training. A separate temporal holdout analysis was also conducted using 2009–2014 for training and 2015–2018 for testing. The resulting performance patterns were compared with those obtained from the base model. Performance was summarized using RMSE, mean absolute error (MAE), and out-of-sample log-likelihood.

## 3 Results

### 3.1 General characteristics

A total of 297,324 malaria cases were analyzed in the Loreto region from 2009 to 2018, in which 79.48% were due to *P. vivax* and 20.52% due to *P. falciparum*. High-transmission season for *P. vivax* occurred between June and August, with the peak of cases in July 2014; while for *P. falciparum,* it was between June and December, with the highest peak in June 2016 (Fig 2). In terms of the cumulative cases of malaria by administrative and hydrobasins areal units, we found the highest mean of *P. vivax* cases (Mean [M] = 151.48, Standard Deviation [SD] = 227.74) and *P. falciparum* cases (M = 39.11; SD = 75.09) in the hydrobasin level 6; and the lowest mean *P. vivax* cases (M = 25.91; SD = 74.07) and *P. falciparum* cases (M = 6.69; SD = 22.58) in the level 7 hydrobasin (Table 1).

**Table 1:** Mean and standard deviation (SD) of the annual malaria cases by parasite species and the annual values of the meteorological and environmental variables in the different administrative and hydrobasin boundaries from 2009 to 2018.

Category	Variable	Geographic boundaries			
		Districts	Hydrobasin level 6	Hydrobasin level 7	Hydrobasin ANA
Malaria cases	*P. Vivax*	37.16 (85.23)	151.48 (227.74)	25.91 (74.07)	59.67 (138.33)
Malaria cases	*P. Falciparum*	9.59 (32.68)	39.11 (75.09)	6.69 (22.58)	15.41 (49.28)
Climatic	Land Surface Temperature	32.56 (1.36)	31.86 (1.72)	32.56 (1.38)	32.52 (1.45)
Climatic	Reference evapotranspiration	84.92 (12.4)	84.15 (11.74)	84.91 (12.37)	85.27 (12.56)
Climatic	Precipitation accumulation	204.67 (86.62)	210.34 (91.6)	212.65 (89.5)	206.58 (86.66)
Climatic	Temperature of the soil in layer (0–7 cm)	25.54 (0.83)	24.62 (1.8)	25.53 (0.88)	25.4 (0.99)
Climatic	Temperature of air at 2m	25.12 (0.83)	24.09 (1.95)	25.09 (0.9)	24.99 (1.02)
Climatic	Minimum temperature	20.4 (2.08)	12.41 (10.28)	20.38 (2.95)	19.94 (2.59)
Climatic	Maximum temperature	31.96 (0.97)	32.2 (0.94)	31.77 (0.9)	31.96 (0.93)
Climatic	Some water from rainfall	14.03 (12.26)	119.88 (92.72)	12.5 (16.95)	22.96 (28.47)
Climatic	Wind speed at 10 m	0.92 (0.21)	1.08 (0.32)	0.96 (0.21)	0.96 (0.2)
Hydrologic	Potential evaporation max	0.01 (0)	0.01 (0.01)	0.01 (0)	0.01 (0.01)
Hydrologic	Potential evaporation min	0 (0)	-0.01 (0)	0 (0)	0 (0)
Hydrologic	Runoff	120.92 (89.18)	128.15 (93.04)	128.7 (92.76)	122.42 (89.24)
Hydrologic	Runoff accumulation	0.12 (0.08)	0.13 (0.07)	0.13 (0.08)	0.13 (0.08)
Hydrologic	Soil Moisture	67.28 (41.95)	67.31 (39.21)	54.81 (32.45)	63.02 (41.13)
Unknown	Actual evapotranspiration	83.72 (11.63)	82.14 (10.88)	83.94 (11.78)	84.14 (11.85)
Unknown	Climate water deficit	1.2 (4.58)	2.01 (5.35)	0.97 (4.33)	1.13 (4.59)
Unknown	Palmer Drought Severity Index	0.16 (2.57)	0.2 (2.4)	0.2 (2.55)	0.48 (2.6)
Vegetation	Enhanced Vegetation Index (EVI)	0.52 (0.05)	0.51 (0.04)	0.51 (0.05)	0.52 (0.05)
Vegetation	Leaf area index high vegetation	5.1 (0.61)	4.97 (0.58)	5.07 (0.59)	5.1 (0.59)
Vegetation	Leaf area index high vegetation max	5.27 (0.62)	5.13 (0.6)	5.24 (0.61)	5.26 (0.6)
Vegetation	Leaf area index high vegetation min	4.93 (0.56)	4.81 (0.53)	4.9 (0.54)	4.94 (0.54)
Vegetation	Leaf area index low vegetation	0.38 (0.29)	0.49 (0.29)	0.47 (0.52)	0.5 (0.42)
Vegetation	Leaf area index low vegetation max	0.38 (0.3)	0.49 (0.29)	0.47 (0.52)	0.5 (0.42)
Vegetation	Leaf area index low vegetation min	0.38 (0.29)	0.49 (0.28)	0.47 (0.52)	0.49 (0.42)
Vegetation	Normalized Difference Vegetation Index (NDVI)	0.81 (0.05)	0.8 (0.05)	0.81 (0.06)	0.81 (0.05)

**Fig 2:**
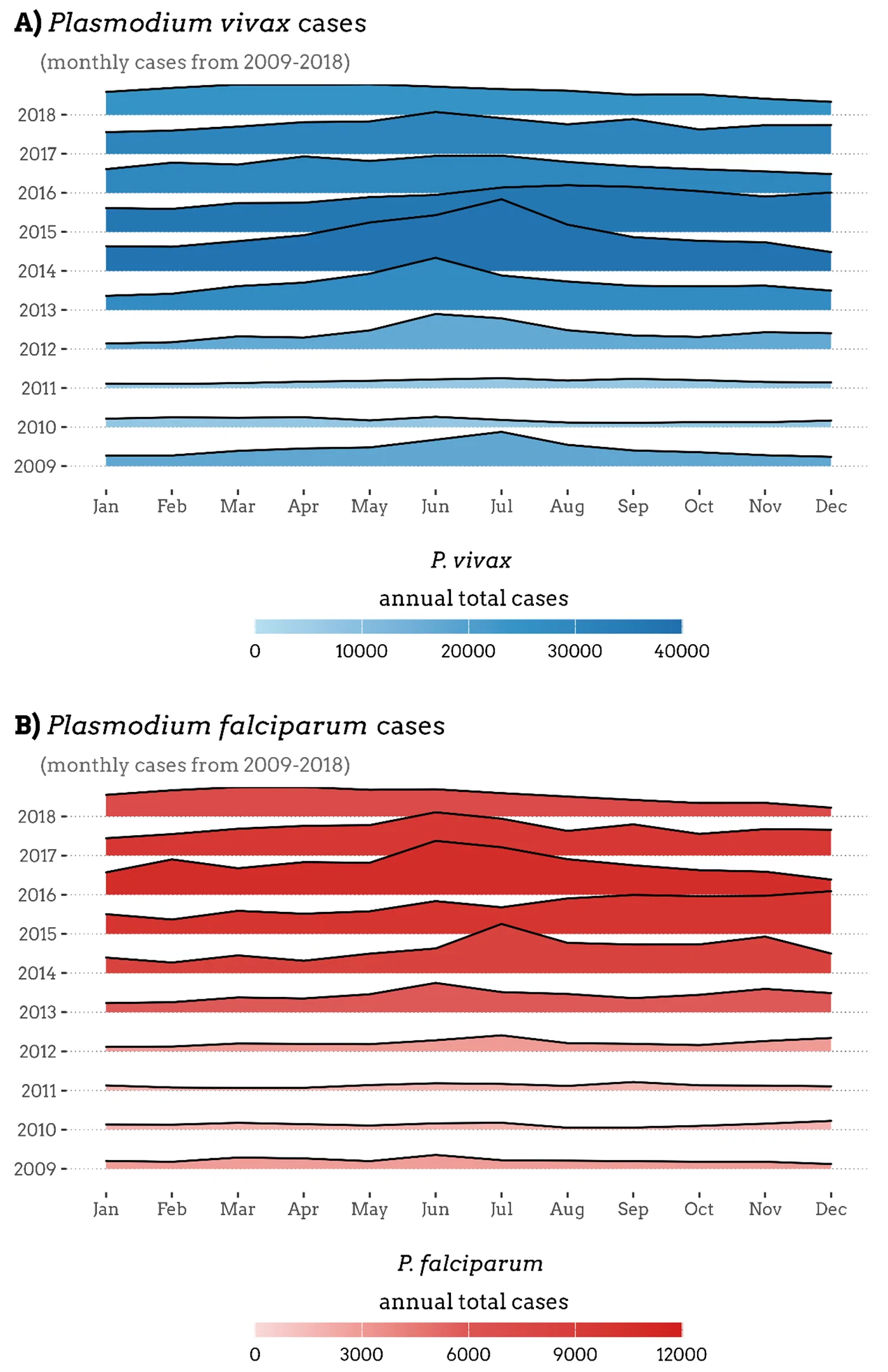
Trend plot to describe the transmission season for both malaria species (number of cases) monthly from 2009 to 2018. This plot was generated with R software v.4.1.2 (R Development Core Team, R Foundation for Statistical Computing, Austria. http://www.R-project.org/).

Regarding the meteorological and environmental factors, the hydrobasin level 6 registered the highest variability levels in most of those factors compared to the rest of boundaries: land surface temperature (M = 31.86 °C; SD = 1.72), precipitation accumulation (M = 210.34; SD = 91.6), temperature of the soil layer (M = 24.62 °C; SD = 1.8), temperature of the air (M = 24.09 °C; SD = 1.95), minimum temperature (M = 12.41 °C; SD = 10.28), some water from rainfall (M = 119.88; SD = 92.72), runoff (M = 128.15; SD = 93.04), and climate water deficit (M = 2.01; SD = 5.35) (Table 1).

Differences in the spatial and demographic structure of the geographic units may also affect how disease and climate signals are aggregated and interpreted. The four delineations examined showed clear differences in these characteristics. Among these, the number of units ranged from 13 level 6 river basins to 76 level 7 river basins. Similarly, the total area covered also varied considerably; level 6 river basins covered the largest area (60,386.6 km^2^), followed by ANA units (11,623.4 km^2^), districts (7,074.4 km^2^) and level 7 river basins (6,186.8 km^2^). Similarly, the average population per spatial unit also varied across the different administrative divisions, being highest in the districts (643 inhabitants) and in the ANA units (557 inhabitants), and lowest in the level 7 catchments (334 inhabitants) (S6 Table).

### 3.2 Modelling and performance metrics

The count-distribution diagnostics supported a negative binomial family with a zero-inflation component (S4 Table). The Poisson models were strongly overdispersed, with Pearson dispersion statistics between 69 and 347, and underpredicted the number of zero counts in every boundary and species. For *P. falciparum* at hydrobasin level 7, for example, 1,591 zero unit-months were expected against 5,785 observed. The NB distribution alone reproduced the observed number of zeros to within 9%. The simulation-based test did not indicate an excess of zeros relative to the NB model in six of eight boundary and species combinations, and in the remaining two (*P. vivax* at ANA and at hydrobasin level 7) fewer zeros were observed than expected. The ZINB model nevertheless had a lower AIC (by 65–1,055 units) and a lower BIC (by 33–1,008 units) than the NB model in all eight combinations. The Vuong test favoured the ZINB model in all eight (z between −16.3 and −4.1, p<0.001). The zero-inflated specification was therefore retained on likelihood-based criteria.

Across all statistical models and for both *P. falciparum* and *P. vivax* species, the hydrobasin level 7 consistently exhibited lower error metrics compared to the district-based and hydrobasin level 6 geometries, as well as the ANA division. This is evidenced by the consistently lower RMSE values observed for this geometry across all statistical models. For instance, for *P. falciparum*, model 1 using hydrobasin level 7 recorded an RMSE of 24.52 (95% CI: 18.78-30.11), lower than the 56.45 (95% CI: 28.79-81.48) and 76.57 (95% CI: 44.40-104.26) observed for the district-based and ANA divisions, respectively. Similarly, for *P. vivax*, model 5 under hydrobasin level 7 yielded an RMSE of 95.43 (95% CI: 57.11-134.23), outperforming the district-based and ANA divisions, which recorded RMSE values of 132.77 (95% CI: 79.37-183.55) and 373.27 (95% CI: 199.94-521.01), respectively (Table 2). Overall, hydrobasin level 7 evidenced the lowest performance metrics compared to the rest of the geographic boundaries in all constructed statistical models.

**Table 2:** Performance metrics of ZINB models for malaria incidence, created based on specific geographic boundaries and species (2009-2018). Four predictors sets were developed for *P. falciparum* (Set 1 optimized for ANA boundaries, Set 2 for District boundaries, Set 3 for hydrobasin level 6, and Set 4 for hydrobasin level 7) and four for *P. vivax* (Set 5 optimized for ANA boundaries, Set 6 for District boundaries, Set 7 for hydrobasin level 6, and Set 8 for hydrobasin level 7).

Geographic boundaries	Predictors sets	Specie	RMSE (95% CI)	AIC
Hydrobasin level 7	Set 1	*P. falciparum*	24.52 (18.78-30.11)	34958
Hydrobasin level 6	Set 1	*P. falciparum*	112.98 (74.61-144.74)	12688
District	Set 1	*P. falciparum*	56.45 (28.79-81.48)	27002
ANA	Set 1	*P. falciparum*	76.57 (44.40-104.26)	17139
Hydrobasin level 7	Set 2	*P. falciparum*	21.80 (16.25-27.07)	34250
Hydrobasin level 6	Set 2	*P. falciparum*	70.79 (40.62-98.52)	12093
District	Set 2	*P. falciparum*	41.67 (20.53-59.57)	29751
ANA	Set 2	*P. falciparum*	59.54 (29.53-83.42)	16414
Hydrobasin level 7	Set 3	*P. falciparum*	22.31 (16.69-27.48)	34513
Hydrobasin level 6	Set 3	*P. falciparum*	75.61 (45.02-100.46)	12242
District	Set 3	*P. falciparum*	70.11 (26.20-111.94)	26686
ANA	Set 3	*P. falciparum*	61.38 (32.72-86.01)	17397
Hydrobasin level 7	Set 4	*P. falciparum*	21.58 (15.97-26.72)	34126
Hydrobasin level 6	Set 4	*P. falciparum*	70.08 (39.79-97.37)	12089
District	Set 4	*P. falciparum*	43.43 (21.02-63.35)	26002
ANA	Set 4	*P. falciparum*	57.64 (28.73-80.36)	16544
Hydrobasin level 7	Set 5	*P. vivax*	95.43 (57.11-134.23)	61196
Hydrobasin level 6	Set 5	*P. vivax*	245.24 (169.91-314.40)	17842
District	Set 5	*P. vivax*	132.77 (79.37-183.55)	46305
ANA	Set 5	*P. vivax*	373.27 (199.94-521.01)	30082
Hydrobasin level 7	Set 6	*P. vivax*	73.67 (45.93-101.81)	60811
Hydrobasin level 6	Set 6	*P. vivax*	273.12 (134.99-413.64)	17625
District	Set 6	*P. vivax*	258.74 (74.21-431.28)	45493
ANA	Set 6	*P. vivax*	155.06 (99.96-205.61)	30469
Hydrobasin level 7	Set 7	*P. vivax*	72.88 (44.12-101.58)	60987
Hydrobasin level 6	Set 7	*P. vivax*	214.12 (122.10-311.74)	17461
District	Set 7	*P. vivax*	244.19 (75.84-403.97)	46074
ANA	Set 7	*P. vivax*	221.18 (94.40-347.15)	30540
Hydrobasin level 7	Set 8	*P. vivax*	72.21 (45.37-99.21)	60724
Hydrobasin level 6	Set 8	*P. vivax*	282.23 (135.96-432.28)	17608
District	Set 8	*P. vivax*	253.59 (73.80-421.81)	45635
ANA	Set 8	*P. vivax*	160.34 (97.04-220.49)	30423

RMSE = Root-Mean-Square Error, AIC = Akaike Information Criterion.

PCA revealed that, in climate components, the highest first component variance was attributed to hydrobasin level 6 analysis (42.2%), while the lowest first component variance was found in hydrobasin ANA (33%). Related to hydrologic components, the highest variance of the first component was found in hydrobasin level 6, and the lowest variance of the first component was found in hydrobasin ANA. Finally, the vegetation component analysis revealed that the highest value of variance in the first component was estimated in hydrobasin level 6, while the lowest variance in the first component was estimated in the district level (41.4%) (S1 Fig).

Fig 3 represented the RMSE estimated values from those models in a radar plot. The inner circle, corresponding to hydrobasin level 7, was the geographic boundary with the lowest RMSE values compared to the rest of boundaries in all performed models by each malaria species. Talking about *P. falciparum* modelling, hydrobasin level 7 evidenced the lowest error metrics in XGBoost (RMSE = 15.79 (95% CI: 10.42-21.03)), random forest (RMSE = 17.21 (95% CI: 11.71-22.54)), support vector machine (RMSE = 19.42 (95% CI: 12.39-25.73)) and ZINB (RMSE = 39.19 (95% CI: 17.09-62.24)) models; while hydrobasin ANA evidenced the highest error metrics in XGBoost (RMSE = 33.15 (95% CI: 16.71-48.81)), random forest (RMSE = 38.00 (95% CI: 19.08-56.82)), support vector machine (RMSE = 45.58 (95% CI: 15.47-71.14)) and ZINB (RMSE = 59.22 (95% CI: 33.26-82.21)) models. Finally, for *P. vivax*, hydrobasin level 7 registered the lowest error metrics in XGBoost (RMSE = 38.44 (95% CI: 26.90-49.19)), random forest (RMSE = 40.98 (95% CI: 28.62-53.08)), support vector machine (RMSE = 46.24 (95% CI: 32.25-59.33)) and ZINB (RMSE = 63.87 (95% CI: 46.38-80.95)) models; while hydrobasin ANA registered higher error metrics in XGBoost (RMSE = 68.99 (95% CI: 45.87-90.45)), random forest (RMSE = 79.10 (95% CI: 50.66-104.94)), support vector machine (RMSE = 95.32 (95% CI: 57.72-128.93)) and ZINB (RMSE = 263.78 (95% CI: 144.98-389.87)) models (S7 Table).

**Fig 3:**
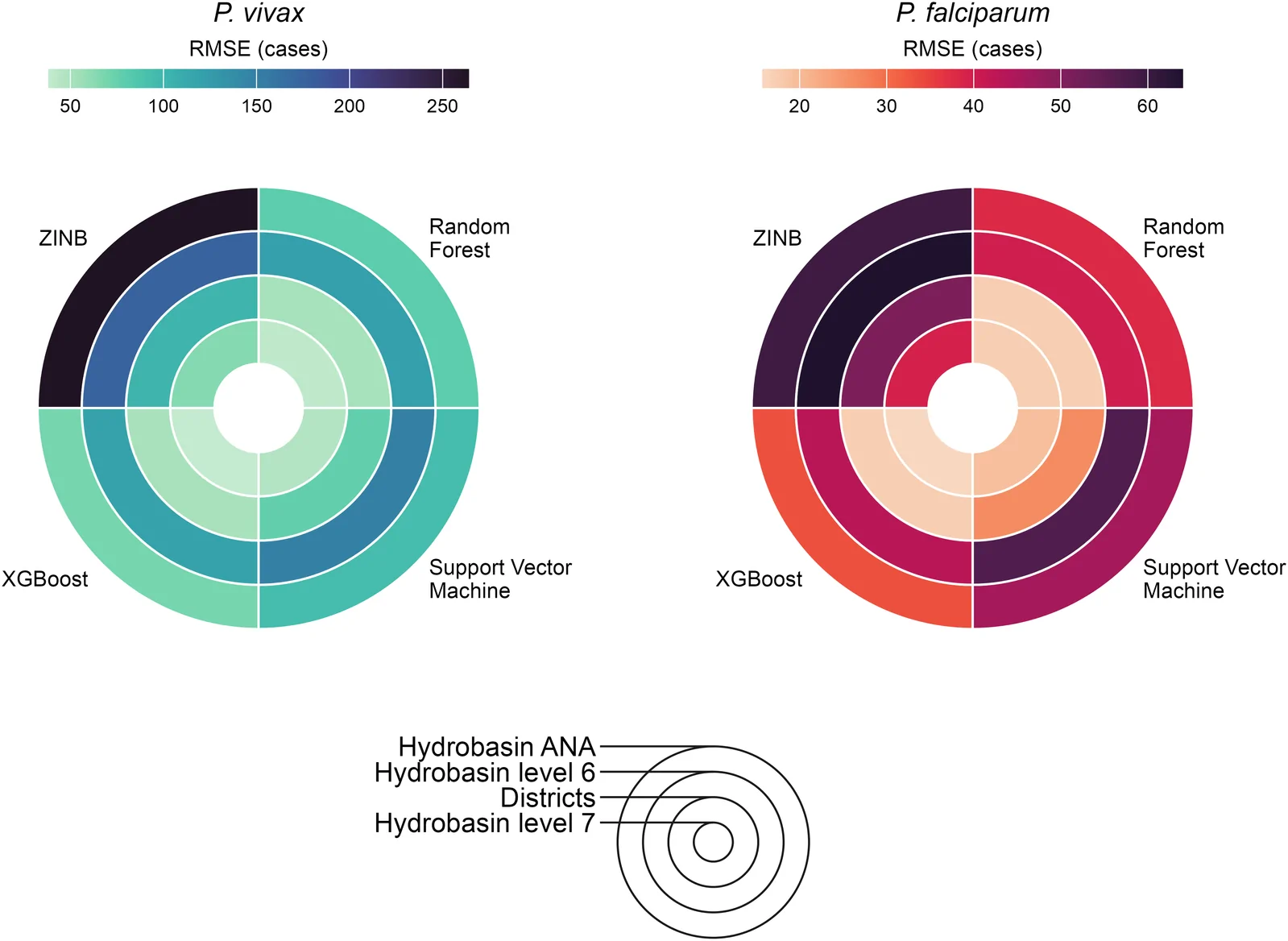
Performance metrics of machine learning models with a PCA-based variable selection method for malaria cases by species and geographic boundaries (2009-2018). The inner circle, the lowest RMSE in that geographic boundary. From the inner to the external circle the order of geographic boundaries is: hydrobasin level 7, District level, hydrobasin level 6, and hydrobasin ANA. Left-side radar plot represents RMSE in *P. vivax* models; while the right-side radar plot represents RMSE in *P. falciparum* models. This plot was generated with R software v.4.1.2 (R Development Core Team, R Foundation for Statistical Computing, Austria. http://www.R-project.org/).

Because RMSE is expressed in cases and therefore depends on the mean count per unit, differences were compared only within a common validation setting. Under the temporal holdout, in which all four boundary systems share the 2009–2014 training and 2015–2018 testing split, hydrobasin level 7 returned the lowest RMSE for both species: 28.67 for *P. falciparum*, 34.5% below districts (43.74), 54.9% below ANA (63.57) and 69.4% below hydrobasin level 6 (93.77); and 73.85 for *P. vivax*, 63.5% below districts (202.55), 64.8% below hydrobasin level 6 (209.74) and 75.1% below ANA (296.77) (S8 Table). Pairwise Diebold-Mariano tests, which compared models evaluated on the same units of a common evaluation boundary, generally supported differences observed in the RMSE-based ranking of predictive performance (S9 Table). However, statistical significance varied across species, evaluation boundaries, and model comparisons, and several models with similar RMSE values did not differ significantly in predictive accuracy.

### 3.3 Residual analysis

Calibration was assessed at the level of the spatial unit. For each unit we computed the mean monthly residual, defined as the observed minus the predicted case count, expressed per 100 inhabitants, and classified units into five categories: strong overestimation (below -0.05 residuals per 100 inhabitants), moderate overestimation (-0.05 to -0.01), well calibrated (-0.01 to 0.01), moderate underestimation (0.01 to 0.05) and strong underestimation (above 0.05) (Fig 4 and S2 Fig). Because each category is a count of a known number of spatial units, proportions are reported with 95% Wilson score confidence intervals (S10 Table). Over-predicting units outnumbered under-predicting units in all eight species by boundary combinations for the ZINB models and in seven of eight for random forest, whereas the support vector machine under-predicted in six of eight and over-predicted in none. XGBoost over-predicted in five of eight combinations and in no case showed net underestimation.

**Fig 4:**
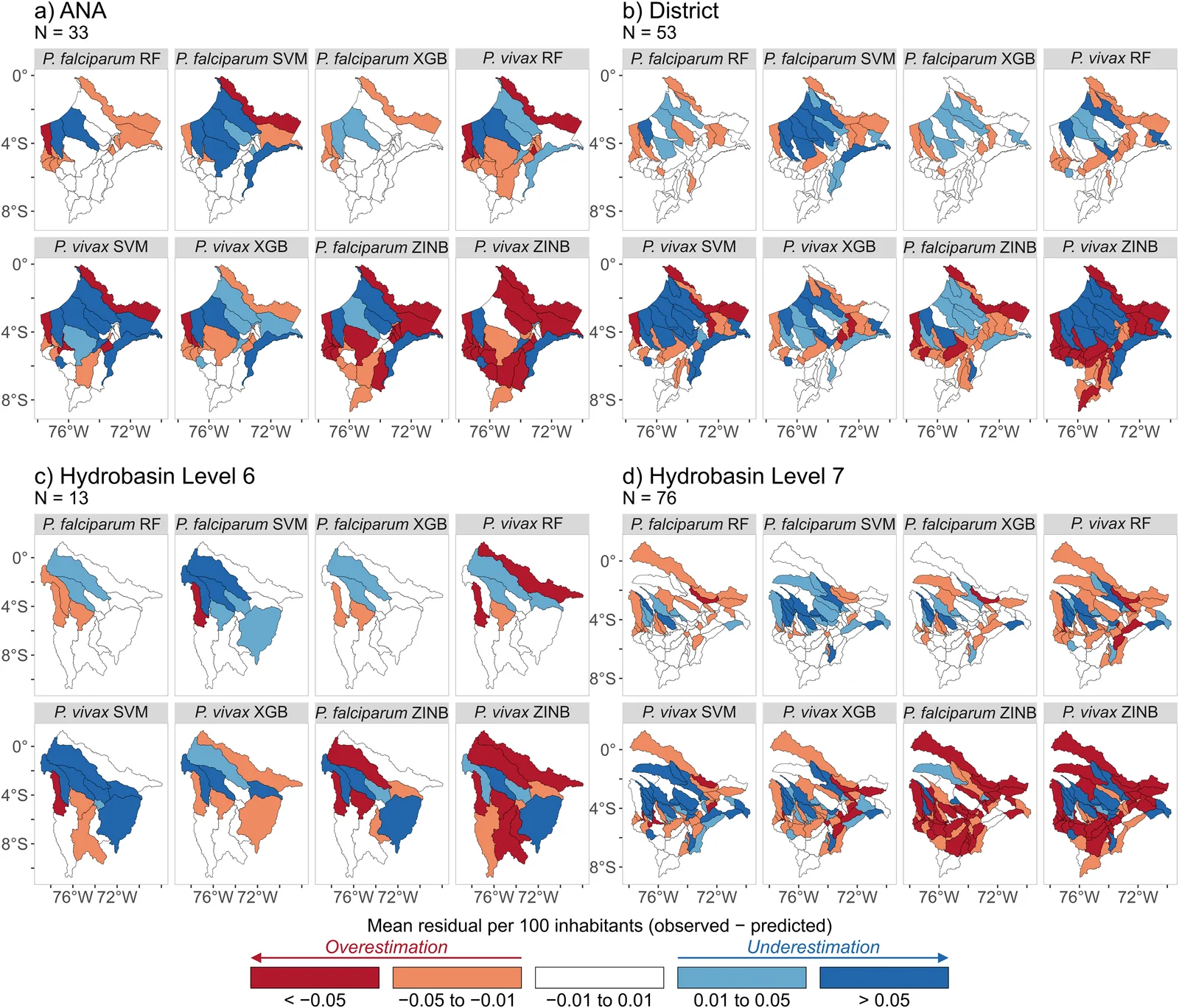
Spatial distribution of mean residuals per 100 inhabitants (observed minus predicted monthly cases) for the ZINB, random forest, support vector machine and XGBoost models, by malaria species and geographic boundary. Units are classified into five categories: strong overestimation (below -0.05 residuals per 100 inhabitants), moderate overestimation (-0.05 to -0.01), well calibrated (-0.01 to 0.01), moderate underestimation (0.01 to 0.05) and strong underestimation (above 0.05). Negative values indicate that the model predicted more cases than were observed. This map plot was generated with R software v.4.1.2 (R Development Core Team, R Foundation for Statistical Computing, Austria. http://www.R-project.org/). Maps were based on public geographic data extracted from INEI, Peru contributors (https://estadist.inei.gob.pe/map) under Open Data Commons Open Database License (ODbL) 1.0 (http://openstreetmap.org/copyright).

For *P. falciparum*, the proportion of well calibrated units, those with residuals within 0.01 per 100 inhabitants of the observed count, ranged from 13.2% (95% CI 7.3 to 22.6) under ZINB at hydrobasin level 7 to 78.8% (95% CI 62.3 to 89.3) under XGBoost at the ANA boundary. Within a fixed boundary, changing the algorithm shifted this proportion by 38.5 to 63.6 percentage points; within a fixed algorithm, changing the boundary shifted it by 14.5 to 20.8 points. The corresponding ranges for *P. vivax* were 18.4 to 53.8 and 12.1 to 34.2 points. Finally, the boundary with the lowest raw error was not the best calibrated on a per-capita scale. Under ZINB for *P. falciparum*, 59.2% of hydrobasin level 7 units (45 of 76; 95% CI 48.0 to 69.6) fell in the strong overestimation category, compared with 13.2% of districts (7 of 53; 95% CI 6.5 to 24.8), even though hydrobasin level 7 returned the lowest RMSE in the same models.

### 3.4 Spatiotemporal cross-validation

Under the blocked spatiotemporal validation framework, predictive performance differed markedly between malaria species and across spatial resolutions (Fig 5). For *P. falciparum*, RMSE values remained relatively low across validation scenarios, ranging from 22.3 to 87.8, with the strongest performance observed when models were trained and evaluated within the same hydrographic scale (e.g., Hydrobasin 07 to Hydrobasin 07 RMSE = 22.3). Cross-scale applications produced moderate increases in prediction error but generally remained below RMSE values of 80. In contrast, *P. vivax* exhibited substantially higher prediction errors and greater variability across spatial contexts. RMSE values ranged from 63.5 to 431.4, with the largest errors occurring when models derived from aggregated hydrobasins were transferred across alternative spatial frameworks (e.g., ANA to ANA RMSE = 431.4). Even the best-performing *P. vivax* models showed errors nearly three times higher than those observed for *P. falciparum*.

**Fig 5:**
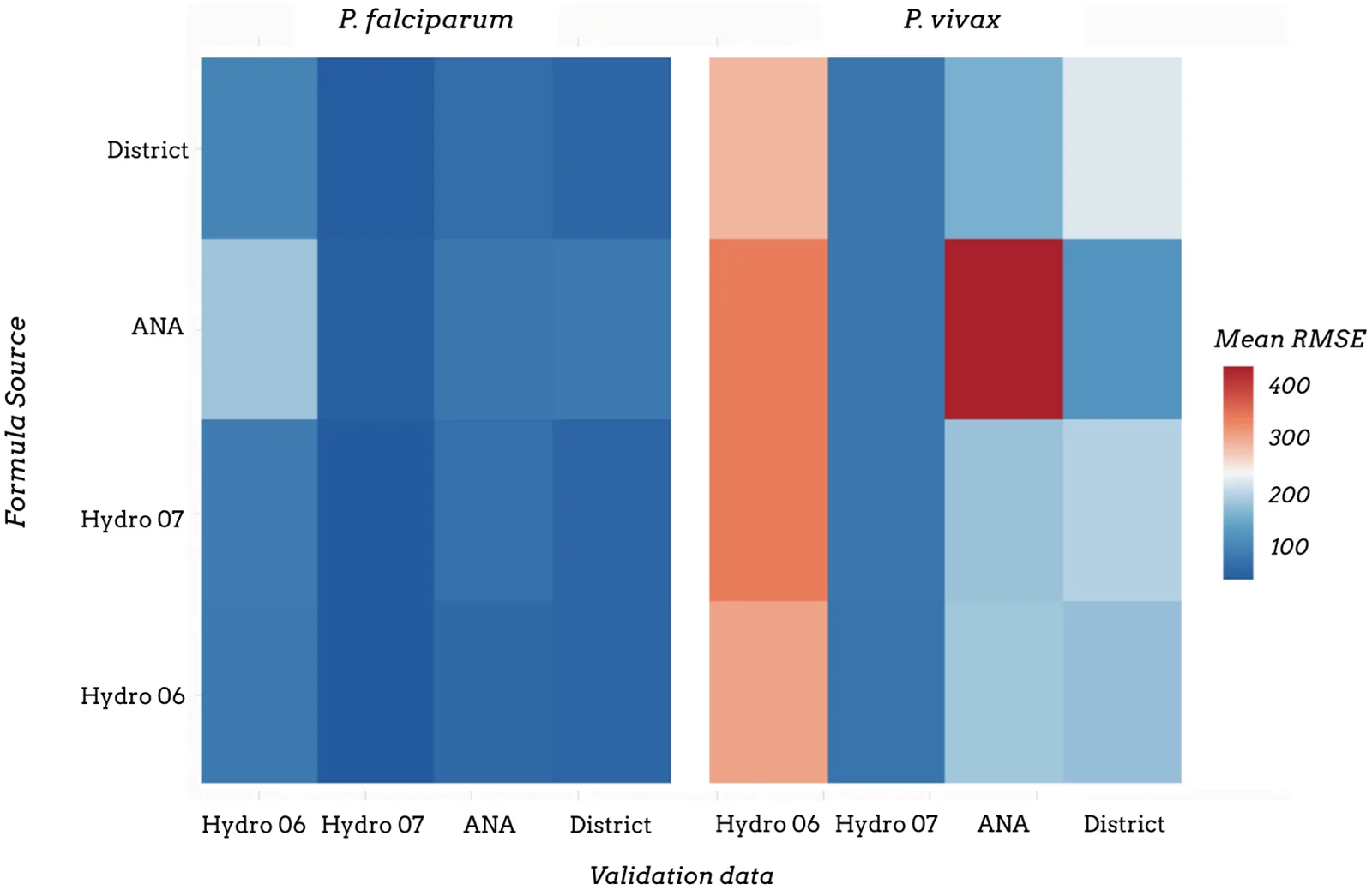
Spatiotemporal transportability of hydrobasin-derived malaria models.

Consistent with these findings, temporal validation results also showed better predictive performance for *P. falciparum* than for *P. vivax* (S8 Table). In the temporal split analysis, *P. falciparum* RMSE values ranged from 28.67 to 93.77 across geographic boundary definitions, whereas *P. vivax* RMSE values ranged from 73.85 to 296.77, indicating persistently greater prediction error for *P. vivax.* The lowest temporal RMSE values were observed at Hydrobasin level 7 for both species (28.67 for *P. falciparum* and 73.85 for *P. vivax*). A similar pattern was observed for MAE, with Hydrobasin level 7 showing the lowest absolute prediction errors for both malaria species (S3 Fig).

## 4 Discussion

Our study used a high spatial resolution of malaria cases to determine the most suitable boundary for malaria case prediction. In this study, we used multiple modelling strategies and covariate-selection processes and found that watershed-based boundaries, particularly hydrobasin level 7, improve malaria prediction accuracy and provide a more ecologically relevant framework for spatial modelling. These results highlight the need for incorporating hydrologically driven spatial units in malaria surveillance and control strategies. With the use of watershed-based boundaries, malaria cases modelling could be improved, helping to better design cost-efficient public health policies and interventions such as distribution of insecticide-treated bed nets, targeting larval source management, or indoor residual spraying, especially in resource-limited settings.

Commonly, malaria modelling studies, which include its climate variables relationship, use administrative boundaries to develop their models. Our study found that watershed-based modelling (i.e., hydrobasins level 7) had the best performance to model the climate-malaria relationship in the Peruvian Amazon. In the riverside localities of the Loreto department, the density of malaria vectors was related to precipitation and river level fluctuation, with behavioral aspects varying between river basins [58]. In fact, surface hydrology is a key factor in determining the abundance of *Anopheles* mosquitoes [59]. By comparing the different models proposed based on climate and environmental variables to explain the dynamics of malaria transmission, we found that the choice of areal unit changed predictive performance materially. This difference may reflect the interaction of climate variables and the hydrological responses that occur within watersheds, as opposed to administrative boundaries, which are usually delimited by political criteria, although the design does not identify the mechanisms involved.

Malaria incidence rates have increased in riverside settlement zones with high economic activity, especially in agriculture [60]. In addition, high entomological inoculation rates have been reported in remote riverine sites in the Peruvian Amazon [61]. Even when outdoor activities that involve human movements among communities from the same hydrobasin mainly condition the high transmissibility in local zones [26], cross-border river network connectivity is an important driver for malaria transmission [62]. Therefore, watershed-based boundaries provide a more realistic ground division approach to model malaria rates.

Watershed-based boundaries can better model the relationship between malaria transmission and climate due to their consideration of landscape topography and the water flow, which can both affect the distribution and behavior of the mosquitoes that transmit malaria. Topography, such as elevation, slope, and aspect, can affect temperature, humidity, and other microclimatic conditions that influence the survival and distribution of mosquitoes. For example, mosquitoes have difficulty surviving in high elevation or steep slope areas [63], and these areas may have a lower risk of malaria transmission. Water flow also plays an important role in the transmission of malaria. Mosquitoes breed in standing water, such as swamps, ponds, and marshes [64]. By using watershed boundaries, researchers can identify regions with more water bodies and, therefore, higher malaria transmission risk. Also, the flow of water can impact the breeding sites for mosquitoes and the potential for malaria transmission.

Another advantage of using watershed-based boundaries is that it allows researchers to identify areas that are likely to be at risk of malaria transmission even in the future. Climate change has led to changes in temperature, precipitation, and water flow, which can, in turn, affect the distribution and behavior of mosquitoes [65,66]. By using watershed-based boundaries, researchers can identify regions that are likely to be at a higher risk of malaria transmission in the future, based on projected changes in climate and water flow [67,68]. Using watershed-based boundaries can provide a more detailed and nuanced understanding of the relationship between malaria transmission and climate by allowing researchers to identify specific areas within a region that are at a higher or lower risk for malaria transmission based on their topography and water flow, and also to predict future risk. However, malaria behavior at a lower level of watersheds, such as Hydrologic Response Units (HRUs), which are small areas that delimit watersheds into homogeneous structures with similar climate, land use, soil and slope characteristics, remains to be explored [27]. HRUs were used in models aimed at predicting the impact of land management and climate change on water resources, working properly [69,70]. We recommend exploring these hydrological units for modelling malaria dynamics.

The spatiotemporal validation showed clear differences between the two malaria species. Prediction errors for *P. falciparum* remained relatively low across spatial scales, whereas *P. vivax* showed higher and more variable errors. This suggests that the relationships captured by the models were more transferable for *P. falciparum*. The lower performance for *P. vivax* may reflect biological and epidemiological features that are not fully represented by the environmental predictors used here, including relapse, broader ecological tolerance, and local variation in human or vector dynamics. The decrease in performance when models were applied across geographic frameworks also shows that results obtained within one set of boundaries may not transfer directly to another. The temporal holdout produced a similar species-specific pattern. Models trained on data from 2009–2014 and evaluated on 2015–2018 generally performed worse than models fitted using the full time series, but *P. falciparum* still showed lower and more stable RMSE values than *P. vivax*. This indicates that some of the predictor–outcome relationships remained useful when applied to later years, particularly for *P. falciparum*. However, differences across species and aggregation levels suggest that model performance should be checked when predictions are extended to new time periods, and recalibration may be needed as transmission patterns change. Model performance also depended on the spatial units used to aggregate the data. Differences in the size and structure of districts and hydrobasins affected the resulting prediction errors, consistent with the spatial support problem in geospatial epidemiology. The choice of spatial boundaries should therefore be considered explicitly when modelling the relationship between environmental conditions and malaria incidence.

We acknowledge some limitations of this study. First, while collapsing data for the entire study period (2009–2018) simplifies our understanding of the relative contribution of spatial boundaries on malaria cases, it is important to recognize the loss of temporal granularity (i.e., from weekly to monthly data). Second, we do not measure migration directly nor account for cross-border malaria importation or human mobility between communities. Carrasco-Escobar *et al*. [71] described that villages with higher connectivity tended to have more malaria cases on the same riverside, which is the basis of boundary segmentation. Third, models evidence an acceptable range of under- and overestimation residual values per inhabitant. However, future studies could include spatial components in the modelling process to better adjust future forecasting strategies. Fourth, the associations between environmental covariates and malaria counts in this study are ecological and were estimated for prediction. They are likely to reflect how covariates contribute to predicting counts in aggregated units and should not be read as causal effects of environmental exposures on individual risk. Finally, previous studies in the Peruvian Amazon [72–74] reported a high number of sub-clinical infections that are not recorded by the MoH during routine data collection. The findings of this study are relevant only for clinical cases, and caution is suggested when interpreting these results for asymptomatic and submicroscopic cases, which can contribute to the maintenance of parasite transmission. Therefore, the incorporation of active-case surveillance data will be crucial to address this limitation in future studies.

Additionally, the advantage of watershed-based units must be read considering the modifiable areal unit problem (MAUP). This problem has two distinct components: a scale effect arising from the number of units, and a zoning effect arising from how a given number of units is composed [35]. The choice of spatial scale has a significant impact on the accuracy of malaria predictive models [75]. For example, an urban malaria model fitted at three nested resolutions predicted best at an intermediate level of aggregation [76]. Malaria risk stratification has itself begun to acknowledge the problem [77]. The zoning component alone is substantial as varying how areal units are combined, with their number held constant, produces more variation in results than varying the number of units does [35]. The same pattern holds in malaria prediction. Holding the number of clusters fixed, partitions drawn to be internally predictable outperformed partitions drawn to be spatially compact, across a range of cluster counts and predictive models [75]. Scale is relevant on the metric as well as on the estimate. Our design varies both components as the four boundary systems differ in their number of units and in their geometry. Fitted to the same case series, the four boundary systems differed in prediction error, and hydrobasin level 7 gave the lowest error in all eight predictor sets. The prediction error is not monotone in granularity; however, a coarser boundary outperformed a finer one in five of the eight modelled sets. This can indicate that the choice of spatial unit affects predictive performance. However, to better establish that hydrological units reduce aggregation bias, studies that hold granularity fixed while varying zoning are necessary.

From a policy perspective, our findings suggest that hydrobasin-based modelling can be integrated into existing administrative decision-making frameworks by serving as an additional layer of risk stratification. While interventions are implemented at the district level, hydrobasin-derived predictions can inform sub-district prioritization, allowing health authorities to allocate resources more efficiently within administrative units. For example, vector control strategies such as larval source management or targeted indoor residual spraying could be concentrated in high-risk hydrological subregions identified within districts.

## 5 Conclusion

Our study demonstrated that watershed-based boundary units, specifically hydrobasin level 7, produced lower prediction errors than traditional administrative divisions for malaria cases in the Peruvian Amazon. By leveraging hydrological divisions, models exhibited consistently lower prediction errors, as evidenced by reduced RMSE values across all statistical and machine learning approaches. These findings highlight the importance of ecological boundaries in understanding malaria dynamics, given their ability to capture the interplay between climatic, hydrological, and environmental factors that influence vector habitats and human mobility. Incorporating watershed-based boundaries into malaria surveillance and control strategies could lead to more precise, geographically targeted interventions, ultimately enhancing public health efforts in endemic regions.

## Supporting information

S1 Fig Principal component analysis of the meteorological and environmental variables with their contributions to each new component and the different watershed area units and administrative limits. (A) Graphic with the two principal components of all variables inside the hydrobasin and administrative limits (B) Graphics with the main principal components in the different limits administrative and watershed area units (districts, level 6 hydrobasin, level 7 hydrobasin and national water authority hydrobasin). This plot was generated with R software v.4.1.2 (R Development Core Team, R Foundation for Statistical Computing, Austria. http://www.R-project.org/).
(TIF)

S2 Fig Proportion of spatial units per model in each residual category, from overestimation to underestimation, by geographic boundary and malaria species. Strong overestimation (below -0.05 residuals per 100 inhabitants), moderate overestimation (-0.05 to -0.01), well calibrated (-0.01 to 0.01), moderate underestimation (0.01 to 0.05) and strong underestimation (above 0.05). This plot was generated with R software v.4.1.2 (R Development Core Team, R Foundation for Statistical Computing, Austria. http://www.R-project.org/).
(TIF)

S3 Fig Mean Absolute Error (MAE) of malaria prediction models across geographic boundary definitions. (TIF)

S1 Table Descriptive characteristics of Hydrobasins levels 5–8 over the study villages of Loreto, compared with the administrative and ANA boundaries, 2009–2018. (DOCX)

S2 Table Meteorological and environmental variables used in the PCA analysis and statistical modelling. (DOCX)

S3 Table Set of predictors used to perform the statistical modelling in each geographic boundary by species under the backward and Elastic Net variable selection methods. (DOCX)

S4 Table Comparative diagnostics of the count distribution by geographic boundary and species. Poisson, negative binomial (NB), zero-inflated Poisson (ZIP) and zero-inflated negative binomial (ZINB) models were fitted with the predictor set selected for each boundary and a log population offset, 2009–2018.
(DOCX)

S5 Table Optimized hyperparameters selected independently for each spatial unit and malaria species. Hyperparameter optimization was performed using random search with five-fold cross-validation and root mean squared error (RMSE) as the selection criterion. mtry, number of predictor variables randomly sampled as candidates at each split in Random Forest; η, learning rate in XGBoost; max_depth, maximum depth of each XGBoost tree; nrounds, number of boosting iterations in XGBoost; C, regularization (penalty) parameter in the support vector machine; σ, bandwidth parameter of the radial basis function kernel in the support vector machine. The number of trees in Random Forest was fixed at ntree = 500 for all models. Search ranges: RF, up to 10 values of mtry sampled at random between 1 and the number of predictors; XGBoost, 20 combinations sampled from η ∈ {0.01, 0.05, 0.10, 0.20, 0.30}, max_depth ∈ {3, 4, 6, 8} and nrounds ∈ {50, 100, 200, 300}, with γ = 0, colsample_bytree = 1, min_child_weight = 1 and subsample = 1 held fixed; SVM with radial basis function kernel, 20 combinations sampled from C ∈ {2^-3^, 2^-2^, …, 2^7^} and σ ∈ {2^-7^, 2^-6^, …, 2^1^}. The same sampled combinations were evaluated for every spatial unit and species.
(DOCX)

S6 Table Number of spatial units, area and mean population by Geographic boundaries (hydrobasins, districts and ANA) Population, cases of malaria (*P. vivax, P. falciparum*), area (km2) and number of spatial units per year, from 2009 to 2018, according to the geographical boundaries analyzed. (DOCX)

S7 Table Performance metrics of machine learning models with a PCA-based variable selection method for malaria cases by species and geographic boundaries (2009–2018). (DOCX)

S8 Table Temporal performance metrics of ZINB models for malaria incidence, stratified by species and geographic boundary definition. The temporal split was defined as 2009–2014 for training and 2015–2018 for testing in order to preserve an adequate number of observations for model training.
(DOCX)

S9 Table Pairwise Diebold-Mariano tests of predictive accuracy. Each test compares two models evaluated on the same boundary (evaluation boundary), using T = 120 monthly differentials of the mean squared error across the units of that boundary. ZINB-X denotes the ZINB model with the predictor set derived in boundary system X and fitted on the evaluation boundary. Variances were estimated with the Newey-West estimator (lag of four months) and the Harvey-Leybourne-Newbold correction.
(DOCX)

S10 Table Distribution of spatial units across residual categories by geographic boundary, malaria species and model, with 95% Wilson score confidence intervals. Residuals are mean monthly observed minus predicted cases per 100 inhabitants. Overestimated combines the strong and moderate overestimation categories (residual below -0.01), underestimated combines the moderate and strong underestimation categories (residual above 0.01).
(DOCX)
