# Supplementary material for: Comparing watershed-based spatial units and administrative boundaries for malaria prediction in the Peruvian Amazon
Source: PLOS Glob Public Health. 2026 Nov 2;6(11):e0007553. doi: 10.1371/journal.pgph.0007553 (PMC13726989; doi:10.1371/journal.pgph.0007553)
Supplement: Supplementary file 1 [file pgph.0007553.s016.pdf]

UNIVERSIDAD PERUANA
CAYETANO HEREDIA

Dear Editor,

We thank you and the reviewers for the evaluation of our manuscript, now titled "*Comparing watershed-based spatial units and administrative boundaries for malaria prediction in the Peruvian Amazon*" (PGPH-D-25-02224R1). The reviewers' comments helped us clarify the central contribution of the study, strengthen the discussion of the Modifiable Areal Unit Problem (MAUP), improve the description of population offsets and boundary construction, and correct inconsistencies in the reporting of model performance and spatial-unit counts.

In the revised manuscript, we have clarified the proposed predictive modeling approach, restrict AIC interpretation to comparisons where likelihoods are comparable, add boundary-level descriptive information to help readers evaluate scale effects, and add additional predictive metrics and uncertainty intervals where feasible. We also corrected textual and reporting issues across the entire document.

Below we provide a point-by-point response to all reviewers' comments and a tracked changes version.

Sincerely,

Gabriel Carrasco Escobar, PhD
Director
Health Innovation Laboratory
Institute of Tropical Medicine "Alexander von Humboldt"
Universidad Peruana Cayetano Heredia

Reviewers' comments

Reviewer 3

1. Authors need to address the MAUP/scale confound directly. Add an explicit discussion of the Modifiable Areal Unit Problem, and ideally a supplementary analysis demonstrating that hydrobasin level 7's RMSE advantage is not simply attributable to it having the smallest average case counts of any boundary type (Table 1).

    - We have incorporated and discussed the MAUP confound. Introduction now defines its two components explicitly, the scale effect arising from the number and size of units and the zoning effect arising from how a fixed number of units is composed, and situates our design against a forecasting literature in which the spatial unit is almost never stated or justified; and the Discussion now carries a dedicated paragraph that separates the two components and relates each to our results. In addition, we also recognize that granularity contributes to this pattern. Hydrobasin level 7 contains the largest number of units and has the lowest mean case count among the boundary systems examined. Because RMSE is expressed in absolute case counts, lower mean counts tend to produce lower RMSE values. Thus, part of the observed ranking likely reflects differences in spatial granularity. We have revised the Discussion accordingly and no longer attribute the full effect to ecological alignment.

2. Revise the cross-boundary AIC comparisons and the cross-application of boundary-specific models to other boundaries' covariate structures (Section 2.4.2/3.2).

    - We agree that AIC should not be interpreted as a direct cross-boundary ranking metric when models are fitted to different response vectors and spatial aggregations. We revised the language so AIC is used only within comparable model/data settings, while cross-boundary evidence is based on out-of-sample predictive metrics (**See section 2.4.4. Performance metrics**).

3. Reconcile the 88%/12% (cited national figure, ref. 5) vs. 79.48%/20.52% (this study's data) P. vivax/P. falciparum split — a brief explanatory sentence is needed.

    - We clarified that the 82%/18% citation refers to national surveillance reporting for Loreto, whereas the 79.48%/20.52% distribution corresponds to the 2009-2018 analytic dataset used in this study.

4. Correct the abstract's percentage-reduction sentences so raw RMSE values and percentage reductions are not conflated in the same clause

    - We corrected the abstract so RMSE values are reported as raw error metrics and relative reductions are only reported when explicitly calculated against a named comparator.

5. Provide the missing total count of ANA hydrobasin units, and consider adding a summary table comparing the four boundary types by number of units, mean unit area, and mean population, to let readers directly assess whether finer resolution alone (rather than hydrological "ecological relevance") explains the result pattern.

    - We have included an additional supplementary table that shows the total number of units by geographic boundaries, the average area, and mean population (**See supplementary table 4**).

6. Reviewer comment: Fix the typo errors. (example : "Lotero" → "Loreto" (line 141), "Hydrostatic Release Units" → "Hydrologic Response Units" (lines 502–503), consistent with your own ref. 27.)

    - We have incorporated these changes in the main text.

7. Fix reference 1's URL (currently points to the 2021, not 2023, report page).

    - We have updated Reference 1, now we are using the 2025 World Malaria Report.

8. Verify in-text citation of references 47 and 48.

    - We verified the placement and relevance of references 47 and 48 citations. References 47 and 48 were included because they provide complementary evidence supporting the biological and ecological rationale for using hydrologically defined spatial units.

Reviewer 4

9. Clarify the population offset: The Methods never state how population was derived. Please specify the source, the allocation method (particularly for the hydrobasins), and whether population varied by year (also for the districts).

    - We have added a Methods sentence specifying the population source, allocation method for hydrobasins, and whether population varied by year. The revised text clarifies that the log population offset was used to model incidence (**See section 2.4.2. Modeling**).

10. Sample size discrepancy: Hydrobasin level 7 is reported as 78 units in the text, 76(Figure 1), and 53 (Figure 4d).

    - We have corrected the sample size across the text and figures.

11. The title contains unnecessary words and, in some cases, unscientific words. I propose that the authors consider revising the title to: "Comparing Watershed-Based Spatial Units and Administrative Boundaries for Malaria Prediction in the Peruvian Amazon".

    - We have changed the title to: "Comparing watershed-based spatial units and administrative boundaries for malaria prediction in the Peruvian Amazon"

Reviewer 5

12. The exact gap is unclear. Stating that watershed-based boundaries are scarce does not make it a gap. Use proper literature synthesis to demonstrate why administrative units produce inferior epidemiological inference by discussing fundamental concepts such as the Modifiable Areal Unit Problem (MAUP), ecological misalignment, spatial aggregation bias, and political versus ecological boundaries. Support your review of existing works with epidemiological theory that discusses ecological fallacy, modifiable areal unit problem (MAUP), and spatial misalignment. Include more forecasting-based literature to support your work. Strengthen the knowledge gap by discussing whether there is evidence in current literature that supports your initial position that current works implicitly assume that administrative units appropriately represent environmentally driven transmission processes, yet this assumption has rarely been empirically evaluated. You may review literature around the interaction of meteorological controls, hydrological controls, and vegetation controls in shaping vector ecology. Briefly summarise previous forecasting frameworks before introducing the spatial-unit problem. Reduce excessive use of statistics in the introduction section and provide a compelling storyline.

    - We have rewritten the Introduction section. We now define the modifiable areal unit problem and both of its components, the scale effect and the zoning effect, and adds

two related concepts that the reviewer identified. In addition, we shortened the descriptive statistics in the opening paragraph and retained only those most relevant to the main argument.

Reviewer #5, methods

13. In your design, explicitly provide clarity by distinguishing between which of the models are for assessing explanatory relationships and which are for predictive modelling. Remove redundancies from the study area write-up. Remove all text that has no methodological relevance to the study objective. For example, do away with information about historical descriptions of the creation of districts and provinces. Provide a concise justification for selecting your three specific scales, such as Hydrobasin level 6, Hydrobasin level 7, and ANA. Explain the rationale behind using different predictor-selection approaches for different modelling families. Why did you use Elastic Net and PCA only for specific models? Provide a metric-based justification for assessing model fit; otherwise, it remains subjective. Shed more light on the Cross-boundary model transfer concept. For ML models, report additional metrics such as MAE, R^2, calibration, and prediction intervals.

- We revised the Methods section to better explain the purpose of each modelling framework, simplify the description of the study area and spatial units, clarify how predictors were selected, and describe model evaluation and cross-boundary transfer in more detail.

  We now distinguish the explanatory and predictive objectives of the modelling approaches in **Section 2.4 (Statistical Analysis)**. The ZINB regression framework was used as a statistical predictive modelling approach appropriate for overdispersed and zero-inflated malaria data. Random Forest, Support Vector Machine, and XGBoost were used as complementary machine learning approaches for prediction, with all models evaluated based on their predictive performance across the different spatial boundaries.

  Section 2.2 (Study Area and Population) and **Section 2.3.3 (Boundary Data)** were modified by removing historical and administrative information that was not relevant. In **Section 2.3.3**, we also added a rationale for the selected spatial scales. Hydrobasin levels 6 and 7 were chosen to provide a coarse-to-fine comparison while maintaining adequate distributions of villages and malaria cases across spatial units. Coarser levels would produce highly aggregated units that could obscure within-basin heterogeneity, whereas finer levels would result in numerous sparsely populated units with few or no malaria cases. The ANA delineation was included because it represents the official hydrological framework used by Peru's National Water Authority, providing a standardized and policy-relevant hydrological boundary for comparison.

We clarified the rationale for the different predictor-selection approaches in **Section 2.4.1 (Predictors Selection)**. For the statistical framework, backward selection and Elastic Net regularization were used to obtain an interpretable predictor set while addressing multicollinearity. PCA was used for the predictive framework to transform correlated environmental predictors into orthogonal components while retaining more than 80% of the original variance.

Section 2.4.4 (Performance Metrics) was updated to provide explicit quantitative criteria for model assessment. RMSE was used as the primary measure of predictive accuracy, whereas AIC was restricted to comparisons among likelihood-based statistical models under comparable response and data settings. RMSE was retained as the primary metric because prediction error on the original malaria-count scale was the main outcome of interest and because RMSE places greater weight on large prediction errors, which are epidemiologically important in high-transmission settings. As a complementary error metric, MAE was additionally evaluated under the more rigorous validation framework described in **Section 2.4.5 (Spatiotemporal Cross-validation)**, with the MAE results reported in **Supplementary Figure 3**.

Finally, we expanded the description of cross-boundary model transfer in **Section 2.4.2 (Modeling)**. Models developed for a given geographic boundary and malaria species were transferred only to alternative geographic boundaries for the same species. This design allowed us to test whether relationships between predictors and outcomes identified under one spatial aggregation could still be applied when the data were organized using a different spatial support.

14. Support your reported statistics with interpretation. Avoid repetition. The result section should report on the residual analysis, explaining specific regions that show systematic over- or underestimation. Quantify clearly the metric difference for comparable validation settings. Identify the most influential predictors. Compute and report 95% confidence intervals for each reported estimate. Provide evidence of statistical comparison by reporting Diebold–Mariano tests or bootstrap comparisons.

- We have revised the Methods and Results sections to provide clearer interpretation and quantitative support for the reported model performance and to reduce repetition.

  We expanded **Section 3.2 (Modeling and Performance Metrics)** to provide interpretation of the reported performance estimates and to quantify differences in

predictive performance across comparable geographic boundaries and modelling approaches.

Section 3.3 residual analysis was also modified to describe the spatial distribution of model overestimation and underestimation across geographic boundaries and malaria species. These spatial patterns are presented in **Figure 4 and Supplementary Figure 2**. Finally, we incorporated the 95% confidence intervals in **Supplementary Table 8.**

We clarified the identification of the most relevant environmental predictors in **Section 2.4.1 (Predictors Selection)**. For the statistical framework, predictors were selected using a combination of backward selection and Elastic Net regularization, with the final predictor sets reported in **Supplementary Table 2**. In parallel, PCA was used to characterize the contribution of climatic, hydrological, and vegetation components, with the corresponding results reported in **Section 3.2 (Modeling and Performance metrics) and Supplementary Figure 1**.

95% confidence intervals for the reported RMSE estimates throughout **Section 3.2 and Supplementary Table 5 have been added**. The uncertainty-estimation procedure is described in **Section 2.4.4 (Performance Metrics)**, where 95% confidence intervals were estimated using 5,000 geographic-unit cluster bootstrap resamples.

Finally, we added formal statistical comparisons of predictive performance using pairwise Diebold-Mariano tests. The statistical procedure is described in **Section 2.4.4 (Performance Metrics)**, including the use of Newey-West variance estimation and the Harvey-Leybourne-Newbold small-sample correction. The corresponding results are reported in **Section 3.2 and Supplementary Table 7**.

15. The authors should directly and explicitly state that their contribution is challenging the assumption that administrative boundaries are the correct spatial unit for infectious disease forecasting. Authors should be able to understand at this point whether their findings state whether their results confirm, contradict, or extend previous prediction research. Discuss MAUP and explain that from your results, different spatial aggregation units alter statistical relationships, and Hydrobasin Level 7 reduces aggregation bias by better aligning with environmental transmission processes. Discuss the observed predictive performance consistency in your Algorithm and geographical location. Avoid speculative inferences.

- We have revised the Discussion to state more clearly that our study challenges the routine use of administrative boundaries as the default spatial unit for malaria forecasting. Our results show that predictive performance changes depending on the spatial aggregation used, with Hydrobasin level 7 consistently showing the lowest prediction errors across several modelling approaches. We now discuss these

findings in relation to the MAUP and previous malaria prediction studies, emphasizing that our results extend previous evidence by showing that both spatial scale and boundary structure can influence prediction. We also avoided claiming that Hydrobasin level 7 definitively reduces aggregation bias, since our design does not isolate zoning from scale effects. Finally, we strengthened the discussion of spatial and temporal validation and limited interpretation to patterns directly supported by the results.
