# Supplementary material for: Comparing watershed-based spatial units and administrative boundaries for malaria prediction in the Peruvian Amazon
Source: PLOS Glob Public Health. 2026 Nov 2;6(11):e0007553. doi: 10.1371/journal.pgph.0007553 (PMC13726989; doi:10.1371/journal.pgph.0007553)
Supplement: Supplementary file 2 [file pgph.0007553.s017.pdf]

Dear Editor,

We thank you and the reviewer for the evaluation of our manuscript "Comparing watershed-based spatial units and administrative boundaries for malaria prediction in the Peruvian Amazon" (PGPH-D-25-02224R2). The comments helped us state the analytical role of each model more precisely, document the diagnostics behind the choice of the count distribution, and clarify how the predictive comparisons were constructed.

In the revised manuscript, we added an epidemiological bridge between the modifiable areal unit problem and malaria transmission ecology, state that the Hydrobasins levels were chosen a priori and document that criterion in a new Supplementary Table 1, and report comparative diagnostics of the count distribution in a new Supplementary Table 4. We also clarified the predictive, non-causal role of the ZINB regression, the hyperparameter search ranges, the construction of the Diebold-Mariano loss differentials and the Newey-West lag, and the meaning of the bootstrap confidence intervals, and we deposited the unit-month analytic datasets in a public repository. Four references were removed with the study-area text and ten methodological references were added, so the reference list was renumbered.

Below we provide a point-by-point response to all comments and a tracked changes version.

Sincerely,

Gabriel Carrasco Escobar, PhD
Director
Health Innovation Laboratory
Institute of Tropical Medicine “Alexander von Humboldt”
Universidad Peruana Cayetano Heredia

Reviewers' comments

1. The authors should strengthen the introduction by providing an epidemiological bridge between spatial-statistical theory and malaria transmission ecology.

    - We have added a paragraph to the Introduction, immediately after the definition of the MAUP, that links the spatial-statistical argument to malaria transmission ecology. It describes how the larval habitats of *Nyssorhynchus darlingi* form through flooding and drainage, and how human exposure in the Amazon is organised along river networks. The paragraph then explains that averaging cases and covariates over units that combine contrasting habitats produces the aggregation bias that underlies the MAUP, and why watersheds, which are delimited by drainage divides, are expected to be more homogeneous than administrative units with respect to the hydrological processes that create breeding habitat. It leads directly to the study hypothesis.

2. Strengthen the Methods section to indicate whether the choice of HydroBASINS Levels 6 and 7 was based on an a priori criterion or an empirical assessment of alternative spatial resolutions.

    - The choice was a priori, and Section 2.3.3 now states this explicitly. Levels 6 and 7 were selected before any model was fitted, on descriptive criteria: the number of units that contained villages, the number of villages per unit and the proportion of unit-months without reported cases. At finer levels many units contained only one village or very few, which made the aggregation unstable, whereas coarser levels grouped the villages into too few units of analysis. To document the criterion, the new Supplementary Table 1 describes Hydrobasins levels 5 to 8 over the study villages. At level 5 the villages fall into only 3 units, whereas at level 8 they fall into 240 units, 56.7% of which contain fewer than five villages, and 77.6% of unit-months have no *P. falciparum* case. No model was fitted at levels 5 or 8.

3. Remove descriptive material concerning territorial hierarchy, governance functions, and institutional organization that does not contribute directly to the analytical design.

    - We have removed this material. In Section 2.2 we deleted the reference to the eight provinces and the paragraph on basic services, poverty, economic activities and the

organization of the regional health system, together with its four references. In Section 2.3.3 we deleted the description of the political and administrative purposes of districts.

4. Retain the information required to define the administrative spatial unit used for aggregation and comparison.

   - We have retained it. Section 2.3.3 now states that districts, the smallest officially recognised administrative subdivision in Peru, nested within provinces and departments, were the administrative unit for aggregation and comparison, and that the 53 official districts of Loreto were used. Section 2.2 keeps the number of districts and refers to this definition.

5. The justification for zero inflation should also be supported by explicit comparative diagnostics rather than the proportion of observed zeros alone.

   - We agree. For each boundary and species, we fitted Poisson, negative binomial (NB), zero-inflated Poisson (ZIP) and ZINB models with the same predictor set and population offset, and compared them with explicit diagnostics (Sections 2.4.2 and 3.2, and the new Supplementary Table 4).

     The NB model alone reproduced the observed number of zeros to within 9%, and a simulation-based test on randomised quantile residuals did not detect excess zeros relative to the NB model in six of eight combinations. The ZINB model nevertheless had a lower AIC (by 65 to 1,055 units) and a lower BIC (by 33 to 1,008 units) than the NB model in all eight combinations, and the Vuong test favoured it in all eight ($p < 0.001$).

     We therefore retained the ZINB on these likelihood-based criteria, and the manuscript now states that the excess of zeros relative to a Poisson distribution arose mainly from overdispersion. The earlier sentence that justified zero inflation by the proportion of zeros and by convergence problems was removed.

6. Clarify whether ZINB is being used for inferential estimation of environmental associations, predictive benchmarking, or both.

   - The ZINB regression was used for predictive benchmarking. Its predictions entered the same RMSE, bootstrap and Diebold-Mariano comparisons as the machine learning models, and it served as the likelihood-based statistical benchmark for those models. It was not used for inferential estimation of environmental associations: its

coefficients are not reported as effect estimates, and no inference is drawn from their statistical significance. Section 2.4 now states this role explicitly.

7. Please make these analytical roles explicit and maintain a non-causal interpretation of regression coefficients.

- Section 2.4 now states that the primary analytical role of every model was predictive and that the ZINB coefficients can be read only as conditional associations at the level of the aggregated spatial unit. Because the predictors were selected by data-driven procedures and the design is ecological and observational, they are not interpreted as causal effects or as individual-level risk factors.

  The same non-causal language was carried into the Abstract, the Discussion, where the difference between boundaries may reflect hydrological processes that the design does not identify, a new limitation on the ecological and predictive nature of the associations, and the Conclusion, which now reports lower prediction errors rather than an ability of watersheds to capture transmission processes.

8. Please report the hyperparameters and tuning ranges for RF, SVM, and XGBoost.

- The tuning ranges are reported in Section 2.4.3, and the selected values for every boundary, species and algorithm in Supplementary Table 5. We have extended the caption of Supplementary Table 5 with the complete search ranges and fixed parameters: for RF, up to 10 values of $mtry$ sampled between 1 and the number of predictors p, with $ntree = 500$; for XGBoost, 20 combinations sampled from $\eta \in \{0.01, 0.05, 0.10, 0.20, 0.30\}$, $max\_depth \in \{3,4,6,8\}$ and $nrounds \in \{50,100,200,300\}$, with $\gamma = 0$, $colsample\_bytree = 1$, $min\_child\_weight = 1$ and $subsample = 1$ held fixed; and for SVM with a radial kernel, 20 combinations sampled from $C \in \{2^{-3}, \dots, 2^{7}\}$ and $\sigma \in \{2^{-7}, \dots, 2^{1}\}$. The same sampled combinations were evaluated for every spatial unit and species, within the training partition.

9. Explain how monthly loss differentials were constructed for Diebold-Mariano tests when the competing boundary systems contain different numbers and configurations of spatial units, and justify the four-month Newey-West lag.

- We added this information in Methods section. Each Diebold-Mariano test compares two models evaluated on the same boundary; no test compares two boundary systems with different units. On a given evaluation boundary, the competing models are the

three machine learning algorithms fitted on that boundary and the four ZINB models, each carrying the predictor set derived in one of the four boundary systems and fitted on the data of the evaluation boundary (the cross-boundary transfer in Section 2.4.2). Both models of a pair therefore predict the same N units in the same months. For month t, the loss differential is the difference between the two models in the mean squared error across these N units, $d_t = \frac{1}{N}\sum_{i=1}^{N} (e_{A,it}^2 - e_{B,it}^2)$, where $e_{A,it}$ and $e_{B,it}$ are the prediction errors of models A and B for unit i in month t. This yields $T = 120$ monthly differentials per comparison. Comparisons between boundary systems with different numbers and configurations of units rest instead on the RMSE, its cluster-bootstrap confidence intervals and the common temporal holdout. The four-month lag was not chosen after inspecting the results. It is the automatic truncation rule of Newey and West (1994), $\lfloor 4(T/100)^{2/9} \rfloor$, which equals four for $T = 120$. The variance of the mean differential uses the Newey-West estimator and the Harvey-Leybourne-Newbold correction, with a Student t reference on $T - 1$ degrees of freedom.

10. Confidence intervals around RMSE quantify uncertainty in a performance statistic and should not be interpreted as prediction intervals for individual malaria outcomes.

- We agree and have added this distinction to Section 2.4.4. The 95% confidence intervals are percentile cluster-bootstrap intervals of the RMSE obtained by resampling spatial units; they describe the uncertainty of a summary performance statistic and are not prediction intervals for the monthly case count of an individual spatial unit. We also corrected the description of the resampling unit, which referred only to hydrobasins although districts were resampled in the same way.
