# Supplementary material for: Comparing watershed-based spatial units and administrative boundaries for malaria prediction in the Peruvian Amazon
Source: PLOS Glob Public Health. 2026 Nov 2;6(11):e0007553. doi: 10.1371/journal.pgph.0007553 (PMC13726989; doi:10.1371/journal.pgph.0007553)
Supplement: Supplementary file 3 [file pgph.0007553.s004.docx]

S1 Table. Descriptive characteristics of Hydrobasins levels 5 to 8 over the study villages of Loreto, compared with the administrative and ANA boundaries, 2009-2018.

| Boundary | Units, n | Mean area, km² | Villages per unit, median (range) | Units with <5 villages, n (%) | Mean annual P. vivax cases per unit | Mean annual P. falciparumcases per unit | Unit-months with no P. vivax case, % | Unit-months with no P. falciparum case, % |
| --- | --- | --- | --- | --- | --- | --- | --- | --- |
| HydroBASINS level 5 | 3 | 409,355 | 659 (132-784) | 0 (0.0) | 7,876.90 | 2,033.90 | 0 | 2.2 |
| HydroBASINS level 6 | 13 | 60,388 | 83 (32-324) | 0 (0.0) | 1,817.70 | 469.4 | 6.7 | 25.3 |
| HydroBASINS level 7 | 76 | 6,187 | 12.5 (1-97) | 15 (19.7) | 310.9 | 80.3 | 35 | 63.4 |
| HydroBASINS level 8 | 240 | 1,124 | 3 (1-54) | 136 (56.7) | 98.5 | 25.4 | 55.5 | 77.6 |
| Districts | 53 | 7,075 | 22 (1-82) | 5 (9.4) | 445.9 | 115.1 | 34.5 | 60.3 |
| ANA watersheds | 33 | 11,624 | 38 (1-197) | 2 (6.1) | 716.1 | 184.9 | 31.5 | 60.7 |
