# Supplementary material for: Comparing watershed-based spatial units and administrative boundaries for malaria prediction in the Peruvian Amazon
Source: PLOS Glob Public Health. 2026 Nov 2;6(11):e0007553. doi: 10.1371/journal.pgph.0007553 (PMC13726989; doi:10.1371/journal.pgph.0007553)
Supplement: Supplementary file 4 [file pgph.0007553.s005.docx]

S2 Table. Meteorological and environmental variables used in the PCA analysis and statistical modelling.

| Category | Variables | Units | Spatial resolution* | Temporal resolution | Source |
| --- | --- | --- | --- | --- | --- |
| Climate | Minimum temperature | °C | 5000 | Monthly | TerraClimate |
| Climate | Maximum temperature | °C | 5000 | Monthly | TerraClimate |
| Climate | Wind speed | m/s | 5000 | Monthly | TerraClimate |
| Climate | Temperature of air at 2m | °K | 11000 | Monthly | ERA5LAND |
| Climate | Temperature of the soil in layer (0-7cm) | °K | 11000 | Monthly | ERA5LAND |
| Climate | Potential evaporation max | m | 11000 | Monthly | ERA5LAND |
| Climate | Potential evaporation min | m | 11000 | Monthly | ERA5LAND |
| Climate | Land Surface Temperature | °K | 1000 | Daily | MYD21C3.061 |
| Climate | Some water from rainfall | m | 11000 | Monthly | ERA5LAND |
| Climate | Accumulated liquid and frozen water, including rain and snow, that falls to the Earth's surface | m | 11000 | Monthly | ERA5LAND |
| Vegetation | Leaf area index high vegetation | Area fraction | 11000 | Monthly | ERA5LAND |
| Vegetation | Leaf area index high vegetation min | Area fraction | 11000 | Monthly | ERA5LAND |
| Vegetation | Leaf area index high vegetation max | Area fraction | 11000 | Monthly | ERA5LAND |
| Vegetation | Leaf area index low vegetation | Area fraction | 11000 | Monthly | ERA5LAND |
| Vegetation | Leaf area index low vegetation min | Area fraction | 11000 | Monthly | ERA5LAND |
| Vegetation | Leaf area index low vegetation max | Area fraction | 11000 | Monthly | ERA5LAND |
| Vegetation | Enhanced Vegetation Index (EVI) | - | 1000 | Monthly | MOD13A3.061 |
| Vegetation | Normalized Difference Vegetation Index (NDVI) | - | 1000 | Monthly | MOD13A3.061 |
| Hydrological | Actual evapotranspiration | mm | 5000 | Monthly | TerraClimate |
| Hydrological | Climate water deficit | mm | 5000 | Monthly | TerraClimate |
| Hydrological | Palmer Drought Severity Index | - | 5000 | Monthly | TerraClimate |
| Hydrological | Reference evapotranspiration | mm | 5000 | Monthly | TerraClimate |
| Hydrological | Precipitation accumulation | mm | 5000 | Monthly | TerraClimate |
| Hydrological | Runoff | mm | 5000 | Monthly | TerraClimate |
| Hydrological | Soil Moisture | mm | 5000 | Monthly | TerraClimate |

*Spatial resolution in meters
