# Supplementary material for: Comparing watershed-based spatial units and administrative boundaries for malaria prediction in the Peruvian Amazon
Source: PLOS Glob Public Health. 2026 Nov 2;6(11):e0007553. doi: 10.1371/journal.pgph.0007553 (PMC13726989; doi:10.1371/journal.pgph.0007553)
Supplement: Supplementary file 5 [file pgph.0007553.s006.docx]

S3 Table. Set of predictors used to perform the statistical modelling in each geographic boundary by species under the backward and Elastic Net variable selection methods.

| Predictors Set | Malaria Species | Variables |
| --- | --- | --- |
| Set 1 | *P. falciparum* | Total precipitation, Palmer drought severity index, Low vegetation leaf area index, High vegetation leaf area index |
| Set 2 | *P. falciparum* | Minimum temperature, Maximum temperature, Low vegetation leaf area index, Total precipitation, Palmer drought severity index, Soil moisture |
| Set 3 | *P. falciparum* | Wind speed, Runoff, Soil moisture, Low vegetation leaf area index, Enhanced vegetation index |
| Set 4 | *P. falciparum* | Wind speed, Total precipitation, Palmer drought severity index, Soil moisture, Low vegetation leaf area index |
| Set 5 | *P. vivax* | Minimum temperature, Wind speed, Total precipitation, Land surface temperature, Low vegetation leaf area index |
| Set 6 | *P. vivax* | Climatic water deficit, Soil moisture, Low vegetation leaf area index, High vegetation leaf area index |
| Set 7 | *P. vivax* | Minimum temperature, Land surface temperature, Climatic water deficit, Soil moisture |
| Set 8 | *P. vivax* | Climatic water deficit, Soil moisture, Low vegetation leaf area index, Normalized difference vegetation index |
