# Supplementary material for: Comparing watershed-based spatial units and administrative boundaries for malaria prediction in the Peruvian Amazon
Source: PLOS Glob Public Health. 2026 Nov 2;6(11):e0007553. doi: 10.1371/journal.pgph.0007553 (PMC13726989; doi:10.1371/journal.pgph.0007553)
Supplement: Supplementary file 6 [file pgph.0007553.s007.docx]

S4 Table. Comparative diagnostics of the count distribution by geographic boundary and species. Poisson, negative binomial (NB), zero-inflated Poisson (ZIP) and zero-inflated negative binomial (ZINB) models were fitted with the predictor set selected for each boundary and a log population offset, 2009-2018.

| Boundary | Species | Set | Observed zeros, n (%) | Expected zeros, Poisson | Expected zeros, NB | Pearson dispersion, Poisson | ΔAIC, NB | ΔBIC, NB | Vuong z | Zero ratio, NB (p) |
| --- | --- | --- | --- | --- | --- | --- | --- | --- | --- | --- |
| ANA | P. falciparum | 1 | 2,403 (60.7) | 687 | 2,419.00 | 207.70 | 519 | 487 | -10.2 | 0.99 (0.588) |
| District | P. falciparum | 2 | 3,832 (60.3) | 1,245 | 3,834.00 | 107.9 | 1,055 | 1,008 | -16.3 | 1.00 (0.932) |
| Hydrobasin L6 | P. falciparum | 3 | 395 (25.3) | 20 | 388.00 | 100.6 | 65 | 33 | -4.1 | 1.02 (0.680) |
| Hydrobasin L7 | P. falciparum | 4 | 5,785 (63.4) | 1,591 | 5,823 | 69.4 | 580 | 537 | -14 | 0.99 (0.416) |
| ANA | P. vivax | 5 | 1,248 (31.5) | 81 | 1,326 | 346.8 | 458 | 420 | -9.9 | 0.94 (0.012) |
| District | P. vivax | 6 | 2,192 (34.5) | 292 | 2,257 | 316.5 | 945 | 912 | -14.6 | 0.97 (0.068) |
| Hydrobasin L6 | P. vivax | 7 | 105 (6.7) | 0 | 96 | 222.1 | 336 | 309 | -7.3 | 1.09 (0.362) |
| Hydrobasin L7 | P. vivax | 8 | 3,194 (35.0) | 237 | 3,298 | 126.7 | 373 | 337 | -8.6 | 0.97 (0.008) |
| ΔAIC and ΔBIC, difference between the NB and the ZINB model fitted to the same data (positive values favour ZINB); AIC and BIC are compared only within a boundary and species. | | | | | | | | | | |
