# Supplementary material for: Comparing watershed-based spatial units and administrative boundaries for malaria prediction in the Peruvian Amazon
Source: PLOS Glob Public Health. 2026 Nov 2;6(11):e0007553. doi: 10.1371/journal.pgph.0007553 (PMC13726989; doi:10.1371/journal.pgph.0007553)
Supplement: Supplementary file 7 [file pgph.0007553.s008.docx]

S5 Table. Optimized hyperparameters selected independently for each spatial unit and malaria species. Hyperparameter optimization was performed using random search with five-fold cross-validation and root mean squared error (RMSE) as the selection criterion. mtry, number of predictor variables randomly sampled as candidates at each split in Random Forest; η, learning rate in XGBoost; max_depth, maximum depth of each XGBoost tree; nrounds, number of boosting iterations in XGBoost; C, regularization (penalty) parameter in the support vector machine; σ, bandwidth parameter of the radial basis function kernel in the support vector machine. The number of trees in Random Forest was fixed at ntree = 500 for all models. Search ranges: RF, up to 10 values of mtry sampled at random between 1 and the number of predictors; XGBoost, 20 combinations sampled from $\boldsymbol{\eta}\boldsymbol{\in\{0.01,0.05,0.10,0.20,0.30\}}$, max_depth $\boldsymbol{\in\{3,4,6,8\}}$ and nrounds $\boldsymbol{\in\{50,100,200,300\}}$, with $\boldsymbol{\gamma}\boldsymbol{=0}$, colsample_bytree = 1, min_child_weight = 1 and subsample = 1 held fixed; SVM with radial basis function kernel, 20 combinations sampled from $\boldsymbol{C}\boldsymbol{\in\{}\boldsymbol{2}^{\boldsymbol{-3}}\boldsymbol{,}\boldsymbol{2}^{\boldsymbol{-2}}\boldsymbol{,\ldots,}\boldsymbol{2}^{\boldsymbol{7}}\boldsymbol{\}}$ and $\boldsymbol{\sigma}\boldsymbol{\in\{}\boldsymbol{2}^{\boldsymbol{-7}}\boldsymbol{,}\boldsymbol{2}^{\boldsymbol{-6}}\boldsymbol{,\ldots,}\boldsymbol{2}^{\boldsymbol{1}}\boldsymbol{\}}$. The same sampled combinations were evaluated for every spatial unit and species.

| Spatial unit | Model | Disease | Parameter | Optimal value |
| --- | --- | --- | --- | --- |
| Districts | RF | *P. falciparum* | mtry | 13 |
| Districts | RF | *P. vivax* | mtry | 12 |
| Districts | XGBoost | *P. falciparum* | η | 0.20 |
| Districts | XGBoost | *P. falciparum* | max_depth | 8 |
| Districts | XGBoost | *P. falciparum* | nrounds | 300 |
| Districts | XGBoost | *P. vivax* | η | 0.20 |
| Districts | XGBoost | *P. vivax* | max_depth | 8 |
| Districts | XGBoost | *P. vivax* | nrounds | 300 |
| Districts | SVM | *P. falciparum* | C | 1 |
| Districts | SVM | *P. falciparum* | σ | 0.250 |
| Districts | SVM | *P. vivax* | C | 64 |
| Districts | SVM | *P. vivax* | σ | 0.500 |
| Hydrobasin L6 | RF | *P. falciparum* | mtry | 10 |
| Hydrobasin L6 | RF | *P. vivax* | mtry | 14 |
| Hydrobasin L6 | XGBoost | *P. falciparum* | η | 0.05 |
| Hydrobasin L6 | XGBoost | *P. falciparum* | max_depth | 4 |
| Hydrobasin L6 | XGBoost | *P. falciparum* | nrounds | 200 |
| Hydrobasin L6 | XGBoost | *P. vivax* | η | 0.20 |
| Hydrobasin L6 | XGBoost | *P. vivax* | max_depth | 8 |
| Hydrobasin L6 | XGBoost | *P. vivax* | nrounds | 300 |
| Hydrobasin L6 | SVM | *P. falciparum* | C | 32 |
| Hydrobasin L6 | SVM | *P. falciparum* | σ | 0.031 |
| Hydrobasin L6 | SVM | *P. vivax* | C | 64 |
| Hydrobasin L6 | SVM | *P. vivax* | σ | 0.031 |
| Hydrobasin L7 | RF | *P. falciparum* | mtry | 11 |
| Hydrobasin L7 | RF | *P. vivax* | mtry | 11 |
| Hydrobasin L7 | XGBoost | *P. falciparum* | η | 0.20 |
| Hydrobasin L7 | XGBoost | *P. falciparum* | max_depth | 8 |
| Hydrobasin L7 | XGBoost | *P. falciparum* | nrounds | 200 |
| Hydrobasin L7 | XGBoost | *P. vivax* | η | 0.20 |
| Hydrobasin L7 | XGBoost | *P. vivax* | max_depth | 8 |
| Hydrobasin L7 | XGBoost | *P. vivax* | nrounds | 300 |
| Hydrobasin L7 | SVM | *P. falciparum* | C | 64 |
| Hydrobasin L7 | SVM | *P. falciparum* | σ | 0.500 |
| Hydrobasin L7 | SVM | *P. vivax* | C | 64 |
| Hydrobasin L7 | SVM | *P. vivax* | σ | 0.250 |
| Hydrobasin ANA | RF | *P. falciparum* | mtry | 4 |
| Hydrobasin ANA | RF | *P. vivax* | mtry | 13 |
| Hydrobasin ANA | XGBoost | *P. falciparum* | η | 0.10 |
| Hydrobasin ANA | XGBoost | *P. falciparum* | max_depth | 3 |
| Hydrobasin ANA | XGBoost | *P. falciparum* | nrounds | 200 |
| Hydrobasin ANA | XGBoost | *P. vivax* | η | 0.30 |
| Hydrobasin ANA | XGBoost | *P. vivax* | max_depth | 6 |
| Hydrobasin ANA | XGBoost | *P. vivax* | nrounds | 200 |
| Hydrobasin ANA | SVM | *P. falciparum* | C | 1 |
| Hydrobasin ANA | SVM | *P. falciparum* | σ | 0.250 |
| Hydrobasin ANA | SVM | *P. vivax* | C | 64 |
| Hydrobasin ANA | SVM | *P. vivax* | σ | 0.031 |
