# Supplementary material for: Comparing watershed-based spatial units and administrative boundaries for malaria prediction in the Peruvian Amazon
Source: PLOS Glob Public Health. 2026 Nov 2;6(11):e0007553. doi: 10.1371/journal.pgph.0007553 (PMC13726989; doi:10.1371/journal.pgph.0007553)
Supplement: Supplementary file 8 [file pgph.0007553.s009.docx]

S6 Table. Number of spatial units, area and mean population by Geographic boundaries (hydrobasins, districts and ANA) Population, cases of malaria (*P. vivax, P. falciparum*), area (km2) and number of spatial units per year, from 2009 to 2018, according to the geographical boundaries analyzed.

| Geographic boundaries | N  units | Area  (km2) | Mean  population |
| --- | --- | --- | --- |
| Hydrobasin level 7 | 76 | 6,186.8 | 334 |
| Hydrobasin level 6 | 13 | 60,386.6 | 404 |
| Districts | 53 | 7,074.4 | 643 |
| ANA | 33 | 11,623.4 | 557 |
