# Supplementary material for: Comparing watershed-based spatial units and administrative boundaries for malaria prediction in the Peruvian Amazon
Source: PLOS Glob Public Health. 2026 Nov 2;6(11):e0007553. doi: 10.1371/journal.pgph.0007553 (PMC13726989; doi:10.1371/journal.pgph.0007553)
Supplement: Supplementary file 9 [file pgph.0007553.s010.docx]

S7 Table. Performance metrics of machine learning models with a PCA-based variable selection method for malaria cases by species and geographic boundaries (2009-2018).

| Geographic boundaries | Machine Learning Model | Specie | RMSE (95% CI) |
| --- | --- | --- | --- |
| Hydrobasin level 7 | XGBoost | *P. falciparum* | 15.79 (10.42-21.03) |
| Districts | XGBoost | *P. falciparum* | 17.10 (11.19-22.57) |
| Hydrobasin level 7 | Random Forest | *P. falciparum* | 17.21 (11.71-22.54) |
| Districts | Random Forest | *P. falciparum* | 17.32 (11.24-22.89) |
| Hydrobasin level 7 | Support Vector Machine | *P. falciparum* | 19.42 (12.39-25.73) |
| Hydrobasin level 7 | ZINB | *P. falciparum* | 39.19 (17.09-62.24) |
| Districts | Support Vector Machine | *P. falciparum* | 26.26 (11.87-39.37) |
| Hydrobasin ANA | XGBoost | *P. falciparum* | 33.15 (16.71-48.81) |
| Districts | ZINB | *P. falciparum* | 50.89 (23.65-77.87) |
| Hydrobasin ANA | Random Forest | *P. falciparum* | 38.00 (19.08-56.82) |
| Hydrobasin level 6 | Random Forest | *P. falciparum* | 39.96 (22.96-54.58) |
| Hydrobasin level 6 | XGBoost | *P. falciparum* | 43.06 (22.93-60.87) |
| Hydrobasin ANA | Support Vector Machine | *P. falciparum* | 45.58 (15.47-71.14) |
| Hydrobasin level 6 | Support Vector Machine | *P. falciparum* | 56.80 (24.27-83.48) |
| Hydrobasin level 6 | ZINB | *P. falciparum* | 63.97 (32.72-89.81) |
| Hydrobasin ANA | ZINB | *P. falciparum* | 59.22 (33.26-82.21) |
| Hydrobasin level 7 | XGBoost | *P. vivax* | 38.44 (26.90-49.19) |
| Hydrobasin level 7 | Random Forest | *P. vivax* | 40.98 (28.62-53.08) |
| Hydrobasin level 7 | Support Vector Machine | *P. vivax* | 46.24 (32.25-59.33) |
| Districts | XGBoost | *P. vivax* | 50.76 (34.16-65.72) |
| Districts | Random Forest | *P. vivax* | 51.84 (32.98-69.06) |
| Hydrobasin ANA | XGBoost | *P. vivax* | 68.99 (45.87-90.45) |
| Districts | Support Vector Machine | *P. vivax* | 76.27 (46.81-102.09) |
| Hydrobasin level 7 | ZINB | *P. vivax* | 63.87 (46.38-80.95) |
| Hydrobasin ANA | Random Forest | *P. vivax* | 79.10 (50.66-104.94) |
| Hydrobasin ANA | Support Vector Machine | *P. vivax* | 95.32 (57.72-128.93) |
| Districts | ZINB | *P. vivax* | 102.81 (73.15-131.77) |
| Hydrobasin level 6 | XGBoost | *P. vivax* | 118.93 (68.50-157.97) |
| Hydrobasin level 6 | Random Forest | *P. vivax* | 122.48 (64.32-168.48) |
| Hydrobasin level 6 | Support Vector Machine | *P. vivax* | 148.68 (77.22-205.99) |
| Hydrobasin ANA | ZINB | *P. vivax* | 263.78 (144.98-389.87) |
| Hydrobasin level 6 | ZINB | *P. vivax* | 175.87 (116.08-232.61) |

RMSE = Root-Mean-Square Error
