# Supplementary material for: Comparing watershed-based spatial units and administrative boundaries for malaria prediction in the Peruvian Amazon
Source: PLOS Glob Public Health. 2026 Nov 2;6(11):e0007553. doi: 10.1371/journal.pgph.0007553 (PMC13726989; doi:10.1371/journal.pgph.0007553)
Supplement: Supplementary file 10 [file pgph.0007553.s011.docx]

S8 Table. Temporal performance metrics of ZINB models for malaria incidence, stratified by species and geographic boundary definition. The temporal split was defined as 2009-2014 for training and 2015-2018 for testing in order to preserve an adequate number of observations for model training.

| Geographic boundaries | Predictors sets | Specie | RMSE | AIC |
| --- | --- | --- | --- | --- |
| Hydrobasin level 6 | Set 3 | *P. falciparum* | 93.77 | 6459.89 |
| Hydrobasin level 7 | Set 4 | *P. falciparum* | 28.67 | 17246.58 |
| District | Set 2 | *P. falciparum* | 43.74 | 13843.42 |
| ANA | Set 1 | *P. falciparum* | 63.57 | 9046.69 |
| Hydrobasin level 6 | Set 7 | *P. vivax* | 209.74 | 9723.14 |
| Hydrobasin level 7 | Set 8 | *P. vivax* | 73.85 | 33728.89 |
| District | Set 6 | *P. vivax* | 202.55 | 25768.14 |
| ANA | Set 5 | *P. vivax* | 296.77 | 16650.05 |

RMSE = Root-Mean-Square Error, AIC = Akaike Information Criterion
