# Supplementary material for: Comparing watershed-based spatial units and administrative boundaries for malaria prediction in the Peruvian Amazon
Source: PLOS Glob Public Health. 2026 Nov 2;6(11):e0007553. doi: 10.1371/journal.pgph.0007553 (PMC13726989; doi:10.1371/journal.pgph.0007553)
Supplement: Supplementary file 11 [file pgph.0007553.s012.docx]

S9 Table. Pairwise Diebold-Mariano tests of predictive accuracy. Each test compares two models evaluated on the same boundary (evaluation boundary), using T=120 monthly differentials of the mean squared error across the units of that boundary. ZINB-X denotes the ZINB model with the predictor set derived in boundary system X and fitted on the evaluation boundary. Variances were estimated with the Newey-West estimator (lag of four months) and the Harvey-Leybourne-Newbold correction.

| Species | Evaluation boundary | Model A | Model B | DM statistic | P value |
| --- | --- | --- | --- | --- | --- |
| *P. falciparum* | District | ZINB-District | ZINB-Hydrobasin 6 | -3.089 | 0.003 |
| *P. falciparum* | District | ZINB-District | ZINB-Hydrobasin 7 | -0.224 | 0.823 |
| *P. falciparum* | District | ZINB-District | ZINB-ANA | -3.723 | <0.001 |
| *P. falciparum* | District | ZINB-District | Random Forest | 4.734 | <0.001 |
| *P. falciparum* | District | ZINB-District | Support Vector Machine | 3.324 | 0.001 |
| *P. falciparum* | District | ZINB-District | XGBoost | 4.729 | <0.001 |
| *P. falciparum* | District | ZINB-Hydrobasin 6 | ZINB-Hydrobasin 7 | 1.5 | 0.136 |
| *P. falciparum* | District | ZINB-Hydrobasin 6 | ZINB-ANA | -2.193 | 0.030 |
| *P. falciparum* | District | ZINB-Hydrobasin 6 | Random Forest | 5.265 | <0.001 |
| *P. falciparum* | District | ZINB-Hydrobasin 6 | Support Vector Machine | 3.821 | <0.001 |
| *P. falciparum* | District | ZINB-Hydrobasin 6 | XGBoost | 5.255 | <0.001 |
| *P. falciparum* | District | ZINB-Hydrobasin 7 | ZINB-ANA | -2.215 | 0.029 |
| *P. falciparum* | District | ZINB-Hydrobasin 7 | Random Forest | 4.744 | <0.001 |
| *P. falciparum* | District | ZINB-Hydrobasin 7 | Support Vector Machine | 4.183 | <0.001 |
| *P. falciparum* | District | ZINB-Hydrobasin 7 | XGBoost | 4.706 | <0.001 |
| *P. falciparum* | District | ZINB-ANA | Random Forest | 5.397 | <0.001 |
| *P. falciparum* | District | ZINB-ANA | Support Vector Machine | 4.111 | <0.001 |
| *P. falciparum* | District | ZINB-ANA | XGBoost | 5.397 | <0.001 |
| *P. falciparum* | District | Random Forest | Support Vector Machine | -2.436 | 0.016 |
| *P. falciparum* | District | Random Forest | XGBoost | 0.469 | 0.640 |
| *P. falciparum* | District | Support Vector Machine | XGBoost | 2.473 | 0.015 |
| *P. vivax* | District | ZINB-District | ZINB-Hydrobasin 6 | 1.85 | 0.067 |
| *P. vivax* | District | ZINB-District | ZINB-Hydrobasin 7 | -5.082 | <0.001 |
| *P. vivax* | District | ZINB-District | ZINB-ANA | 0.546 | 0.586 |
| *P. vivax* | District | ZINB-District | Random Forest | 8.788 | <0.001 |
| *P. vivax* | District | ZINB-District | Support Vector Machine | 5.608 | <0.001 |
| *P. vivax* | District | ZINB-District | XGBoost | 8.605 | <0.001 |
| *P. vivax* | District | ZINB-Hydrobasin 6 | ZINB-Hydrobasin 7 | -4.843 | <0.001 |
| *P. vivax* | District | ZINB-Hydrobasin 6 | ZINB-ANA | -1.253 | 0.213 |
| *P. vivax* | District | ZINB-Hydrobasin 6 | Random Forest | 10.056 | <0.001 |
| *P. vivax* | District | ZINB-Hydrobasin 6 | Support Vector Machine | 6.262 | <0.001 |
| *P. vivax* | District | ZINB-Hydrobasin 6 | XGBoost | 9.554 | <0.001 |
| *P. vivax* | District | ZINB-Hydrobasin 7 | ZINB-ANA | 5.36 | <0.001 |
| *P. vivax* | District | ZINB-Hydrobasin 7 | Random Forest | 8.355 | <0.001 |
| *P. vivax* | District | ZINB-Hydrobasin 7 | Support Vector Machine | 6.613 | <0.001 |
| *P. vivax* | District | ZINB-Hydrobasin 7 | XGBoost | 8.328 | <0.001 |
| *P. vivax* | District | ZINB-ANA | Random Forest | 8.412 | <0.001 |
| *P. vivax* | District | ZINB-ANA | Support Vector Machine | 4.971 | <0.001 |
| *P. vivax* | District | ZINB-ANA | XGBoost | 8.252 | <0.001 |
| *P. vivax* | District | Random Forest | Support Vector Machine | -3.894 | <0.001 |
| *P. vivax* | District | Random Forest | XGBoost | 0.314 | 0.754 |
| *P. vivax* | District | Support Vector Machine | XGBoost | 3.883 | <0.001 |
| *P. falciparum* | Hydrobasin ANA | ZINB-District | ZINB-Hydrobasin 6 | -1.397 | 0.165 |
| *P. falciparum* | Hydrobasin ANA | ZINB-District | ZINB-Hydrobasin 7 | 2.295 | 0.023 |
| *P. falciparum* | Hydrobasin ANA | ZINB-District | ZINB-ANA | 2.318 | 0.022 |
| *P. falciparum* | Hydrobasin ANA | ZINB-District | Random Forest | 4.179 | <0.001 |
| *P. falciparum* | Hydrobasin ANA | ZINB-District | Support Vector Machine | 3.922 | <0.001 |
| *P. falciparum* | Hydrobasin ANA | ZINB-District | XGBoost | 4.285 | <0.001 |
| *P. falciparum* | Hydrobasin ANA | ZINB-Hydrobasin 6 | ZINB-Hydrobasin 7 | 2.598 | 0.011 |
| *P. falciparum* | Hydrobasin ANA | ZINB-Hydrobasin 6 | ZINB-ANA | 2.81 | 0.006 |
| *P. falciparum* | Hydrobasin ANA | ZINB-Hydrobasin 6 | Random Forest | 4.255 | <0.001 |
| *P. falciparum* | Hydrobasin ANA | ZINB-Hydrobasin 6 | Support Vector Machine | 4.052 | <0.001 |
| *P. falciparum* | Hydrobasin ANA | ZINB-Hydrobasin 6 | XGBoost | 4.333 | <0.001 |
| *P. falciparum* | Hydrobasin ANA | ZINB-Hydrobasin 7 | ZINB-ANA | -1.168 | 0.245 |
| *P. falciparum* | Hydrobasin ANA | ZINB-Hydrobasin 7 | Random Forest | 2.908 | 0.004 |
| *P. falciparum* | Hydrobasin ANA | ZINB-Hydrobasin 7 | Support Vector Machine | 2.471 | 0.015 |
| *P. falciparum* | Hydrobasin ANA | ZINB-Hydrobasin 7 | XGBoost | 3.109 | 0.002 |
| *P. falciparum* | Hydrobasin ANA | ZINB-ANA | Random Forest | 4.658 | <0.001 |
| *P. falciparum* | Hydrobasin ANA | ZINB-ANA | Support Vector Machine | 4.232 | <0.001 |
| *P. falciparum* | Hydrobasin ANA | ZINB-ANA | XGBoost | 4.78 | <0.001 |
| *P. falciparum* | Hydrobasin ANA | Random Forest | Support Vector Machine | -2.443 | 0.016 |
| *P. falciparum* | Hydrobasin ANA | Random Forest | XGBoost | 3.045 | 0.003 |
| *P. falciparum* | Hydrobasin ANA | Support Vector Machine | XGBoost | 2.938 | 0.004 |
| *P. vivax* | Hydrobasin ANA | ZINB-District | ZINB-Hydrobasin 6 | -0.98 | 0.329 |
| *P. vivax* | Hydrobasin ANA | ZINB-District | ZINB-Hydrobasin 7 | -1.077 | 0.284 |
| *P. vivax* | Hydrobasin ANA | ZINB-District | ZINB-ANA | -1.835 | 0.069 |
| *P. vivax* | Hydrobasin ANA | ZINB-District | Random Forest | 3.795 | <0.001 |
| *P. vivax* | Hydrobasin ANA | ZINB-District | Support Vector Machine | 3.672 | <0.001 |
| *P. vivax* | Hydrobasin ANA | ZINB-District | XGBoost | 3.924 | <0.001 |
| *P. vivax* | Hydrobasin ANA | ZINB-Hydrobasin 6 | ZINB-Hydrobasin 7 | -0.868 | 0.387 |
| *P. vivax* | Hydrobasin ANA | ZINB-Hydrobasin 6 | ZINB-ANA | 0.097 | 0.923 |
| *P. vivax* | Hydrobasin ANA | ZINB-Hydrobasin 6 | Random Forest | 3.317 | 0.001 |
| *P. vivax* | Hydrobasin ANA | ZINB-Hydrobasin 6 | Support Vector Machine | 3.201 | 0.002 |
| *P. vivax* | Hydrobasin ANA | ZINB-Hydrobasin 6 | XGBoost | 3.418 | <0.001 |
| *P. vivax* | Hydrobasin ANA | ZINB-Hydrobasin 7 | ZINB-ANA | 0.954 | 0.342 |
| *P. vivax* | Hydrobasin ANA | ZINB-Hydrobasin 7 | Random Forest | 2.261 | 0.026 |
| *P. vivax* | Hydrobasin ANA | ZINB-Hydrobasin 7 | Support Vector Machine | 2.151 | 0.034 |
| *P. vivax* | Hydrobasin ANA | ZINB-Hydrobasin 7 | XGBoost | 2.319 | 0.022 |
| *P. vivax* | Hydrobasin ANA | ZINB-ANA | Random Forest | 3.531 | <0.001 |
| *P. vivax* | Hydrobasin ANA | ZINB-ANA | Support Vector Machine | 3.422 | <0.001 |
| *P. vivax* | Hydrobasin ANA | ZINB-ANA | XGBoost | 3.642 | <0.001 |
| *P. vivax* | Hydrobasin ANA | Random Forest | Support Vector Machine | -3.225 | 0.002 |
| *P. vivax* | Hydrobasin ANA | Random Forest | XGBoost | 2.382 | 0.019 |
| *P. vivax* | Hydrobasin ANA | Support Vector Machine | XGBoost | 3.463 | <0.001 |
| *P. falciparum* | Hydrobasin level 6 | ZINB-District | ZINB-Hydrobasin 6 | -1.018 | 0.311 |
| *P. falciparum* | Hydrobasin level 6 | ZINB-District | ZINB-Hydrobasin 7 | -1.018 | 0.311 |
| *P. falciparum* | Hydrobasin level 6 | ZINB-District | ZINB-ANA | 1.018 | 0.311 |
| *P. falciparum* | Hydrobasin level 6 | ZINB-District | Random Forest | 1.018 | 0.311 |
| *P. falciparum* | Hydrobasin level 6 | ZINB-District | Support Vector Machine | 1.018 | 0.311 |
| *P. falciparum* | Hydrobasin level 6 | ZINB-District | XGBoost | 1.018 | 0.311 |
| *P. falciparum* | Hydrobasin level 6 | ZINB-Hydrobasin 6 | ZINB-Hydrobasin 7 | -1.018 | 0.311 |
| *P. falciparum* | Hydrobasin level 6 | ZINB-Hydrobasin 6 | ZINB-ANA | 1.018 | 0.311 |
| *P. falciparum* | Hydrobasin level 6 | ZINB-Hydrobasin 6 | Random Forest | 1.018 | 0.311 |
| *P. falciparum* | Hydrobasin level 6 | ZINB-Hydrobasin 6 | Support Vector Machine | 1.018 | 0.311 |
| *P. falciparum* | Hydrobasin level 6 | ZINB-Hydrobasin 6 | XGBoost | 1.018 | 0.311 |
| *P. falciparum* | Hydrobasin level 6 | ZINB-Hydrobasin 7 | ZINB-ANA | 1.018 | 0.311 |
| *P. falciparum* | Hydrobasin level 6 | ZINB-Hydrobasin 7 | Random Forest | 1.018 | 0.311 |
| *P. falciparum* | Hydrobasin level 6 | ZINB-Hydrobasin 7 | Support Vector Machine | 1.018 | 0.311 |
| *P. falciparum* | Hydrobasin level 6 | ZINB-Hydrobasin 7 | XGBoost | 1.018 | 0.311 |
| *P. falciparum* | Hydrobasin level 6 | ZINB-ANA | Random Forest | 1.02 | 0.310 |
| *P. falciparum* | Hydrobasin level 6 | ZINB-ANA | Support Vector Machine | 1.019 | 0.310 |
| *P. falciparum* | Hydrobasin level 6 | ZINB-ANA | XGBoost | 1.02 | 0.310 |
| *P. falciparum* | Hydrobasin level 6 | Random Forest | Support Vector Machine | -2.091 | 0.039 |
| *P. falciparum* | Hydrobasin level 6 | Random Forest | XGBoost | -0.818 | 0.415 |
| *P. falciparum* | Hydrobasin level 6 | Support Vector Machine | XGBoost | 2.139 | 0.035 |
| *P. vivax* | Hydrobasin level 6 | ZINB-District | ZINB-Hydrobasin 6 | 1.04 | 0.300 |
| *P. vivax* | Hydrobasin level 6 | ZINB-District | ZINB-Hydrobasin 7 | -1.003 | 0.318 |
| *P. vivax* | Hydrobasin level 6 | ZINB-District | ZINB-ANA | 1.02 | 0.310 |
| *P. vivax* | Hydrobasin level 6 | ZINB-District | Random Forest | 1.069 | 0.287 |
| *P. vivax* | Hydrobasin level 6 | ZINB-District | Support Vector Machine | 1.051 | 0.295 |
| *P. vivax* | Hydrobasin level 6 | ZINB-District | XGBoost | 1.073 | 0.285 |
| *P. vivax* | Hydrobasin level 6 | ZINB-Hydrobasin 6 | ZINB-Hydrobasin 7 | -1.02 | 0.310 |
| *P. vivax* | Hydrobasin level 6 | ZINB-Hydrobasin 6 | ZINB-ANA | -1.123 | 0.264 |
| *P. vivax* | Hydrobasin level 6 | ZINB-Hydrobasin 6 | Random Forest | 1.702 | 0.092 |
| *P. vivax* | Hydrobasin level 6 | ZINB-Hydrobasin 6 | Support Vector Machine | 1.216 | 0.226 |
| *P. vivax* | Hydrobasin level 6 | ZINB-Hydrobasin 6 | XGBoost | 1.86 | 0.065 |
| *P. vivax* | Hydrobasin level 6 | ZINB-Hydrobasin 7 | ZINB-ANA | 1.01 | 0.315 |
| *P. vivax* | Hydrobasin level 6 | ZINB-Hydrobasin 7 | Random Forest | 1.034 | 0.303 |
| *P. vivax* | Hydrobasin level 6 | ZINB-Hydrobasin 7 | Support Vector Machine | 1.026 | 0.307 |
| *P. vivax* | Hydrobasin level 6 | ZINB-Hydrobasin 7 | XGBoost | 1.036 | 0.302 |
| *P. vivax* | Hydrobasin level 6 | ZINB-ANA | Random Forest | 1.235 | 0.219 |
| *P. vivax* | Hydrobasin level 6 | ZINB-ANA | Support Vector Machine | 1.158 | 0.249 |
| *P. vivax* | Hydrobasin level 6 | ZINB-ANA | XGBoost | 1.256 | 0.212 |
| *P. vivax* | Hydrobasin level 6 | Random Forest | Support Vector Machine | -1.832 | 0.070 |
| *P. vivax* | Hydrobasin level 6 | Random Forest | XGBoost | 1.02 | 0.310 |
| *P. vivax* | Hydrobasin level 6 | Support Vector Machine | XGBoost | 1.962 | 0.052 |
| *P. falciparum* | Hydrobasin level 7 | ZINB-District | ZINB-Hydrobasin 6 | -0.123 | 0.902 |
| *P. falciparum* | Hydrobasin level 7 | ZINB-District | ZINB-Hydrobasin 7 | -0.433 | 0.666 |
| *P. falciparum* | Hydrobasin level 7 | ZINB-District | ZINB-ANA | -1.167 | 0.246 |
| *P. falciparum* | Hydrobasin level 7 | ZINB-District | Random Forest | 3.94 | <0.001 |
| *P. falciparum* | Hydrobasin level 7 | ZINB-District | Support Vector Machine | 0.938 | 0.350 |
| *P. falciparum* | Hydrobasin level 7 | ZINB-District | XGBoost | 6.724 | <0.001 |
| *P. falciparum* | Hydrobasin level 7 | ZINB-Hydrobasin 6 | ZINB-Hydrobasin 7 | -0.181 | 0.857 |
| *P. falciparum* | Hydrobasin level 7 | ZINB-Hydrobasin 6 | ZINB-ANA | -0.977 | 0.331 |
| *P. falciparum* | Hydrobasin level 7 | ZINB-Hydrobasin 6 | Random Forest | 3.646 | <0.001 |
| *P. falciparum* | Hydrobasin level 7 | ZINB-Hydrobasin 6 | Support Vector Machine | 0.872 | 0.385 |
| *P. falciparum* | Hydrobasin level 7 | ZINB-Hydrobasin 6 | XGBoost | 6.165 | <0.001 |
| *P. falciparum* | Hydrobasin level 7 | ZINB-Hydrobasin 7 | ZINB-ANA | -0.809 | 0.420 |
| *P. falciparum* | Hydrobasin level 7 | ZINB-Hydrobasin 7 | Random Forest | 4.444 | <0.001 |
| *P. falciparum* | Hydrobasin level 7 | ZINB-Hydrobasin 7 | Support Vector Machine | 0.985 | 0.326 |
| *P. falciparum* | Hydrobasin level 7 | ZINB-Hydrobasin 7 | XGBoost | 7.484 | <0.001 |
| *P. falciparum* | Hydrobasin level 7 | ZINB-ANA | Random Forest | 3.952 | <0.001 |
| *P. falciparum* | Hydrobasin level 7 | ZINB-ANA | Support Vector Machine | 1.131 | 0.260 |
| *P. falciparum* | Hydrobasin level 7 | ZINB-ANA | XGBoost | 5.996 | <0.001 |
| *P. falciparum* | Hydrobasin level 7 | Random Forest | Support Vector Machine | -2.248 | 0.026 |
| *P. falciparum* | Hydrobasin level 7 | Random Forest | XGBoost | 2.615 | 0.010 |
| *P. falciparum* | Hydrobasin level 7 | Support Vector Machine | XGBoost | 3.333 | 0.001 |
| *P. vivax* | Hydrobasin level 7 | ZINB-District | ZINB-Hydrobasin 6 | 2.153 | 0.033 |
| *P. vivax* | Hydrobasin level 7 | ZINB-District | ZINB-Hydrobasin 7 | 1.95 | 0.053 |
| *P. vivax* | Hydrobasin level 7 | ZINB-District | ZINB-ANA | 0.997 | 0.321 |
| *P. vivax* | Hydrobasin level 7 | ZINB-District | Random Forest | 5.644 | <0.001 |
| *P. vivax* | Hydrobasin level 7 | ZINB-District | Support Vector Machine | 5.13 | <0.001 |
| *P. vivax* | Hydrobasin level 7 | ZINB-District | XGBoost | 5.491 | <0.001 |
| *P. vivax* | Hydrobasin level 7 | ZINB-Hydrobasin 6 | ZINB-Hydrobasin 7 | -1.365 | 0.175 |
| *P. vivax* | Hydrobasin level 7 | ZINB-Hydrobasin 6 | ZINB-ANA | -2.114 | 0.037 |
| *P. vivax* | Hydrobasin level 7 | ZINB-Hydrobasin 6 | Random Forest | 5.594 | <0.001 |
| *P. vivax* | Hydrobasin level 7 | ZINB-Hydrobasin 6 | Support Vector Machine | 5.192 | <0.001 |
| *P. vivax* | Hydrobasin level 7 | ZINB-Hydrobasin 6 | XGBoost | 5.446 | <0.001 |
| *P. vivax* | Hydrobasin level 7 | ZINB-Hydrobasin 7 | ZINB-ANA | -1.71 | 0.090 |
| *P. vivax* | Hydrobasin level 7 | ZINB-Hydrobasin 7 | Random Forest | 5.561 | <0.001 |
| *P. vivax* | Hydrobasin level 7 | ZINB-Hydrobasin 7 | Support Vector Machine | 5.209 | <0.001 |
| *P. vivax* | Hydrobasin level 7 | ZINB-Hydrobasin 7 | XGBoost | 5.29 | <0.001 |
| *P. vivax* | Hydrobasin level 7 | ZINB-ANA | Random Forest | 5.866 | <0.001 |
| *P. vivax* | Hydrobasin level 7 | ZINB-ANA | Support Vector Machine | 5.087 | <0.001 |
| *P. vivax* | Hydrobasin level 7 | ZINB-ANA | XGBoost | 5.631 | <0.001 |
| *P. vivax* | Hydrobasin level 7 | Random Forest | Support Vector Machine | -2.056 | 0.042 |
| *P. vivax* | Hydrobasin level 7 | Random Forest | XGBoost | 1.312 | 0.192 |
| *P. vivax* | Hydrobasin level 7 | Support Vector Machine | XGBoost | 2.288 | 0.024 |
| Abbreviations: ZINB, Zero-Inflated Negative Binomial; DM, Diebold-Mariano. Bold P values indicate statistical significance at P<0.05. | | | | | |
|  |  |  |  |  |  |
