# Supplementary material for: Comparing watershed-based spatial units and administrative boundaries for malaria prediction in the Peruvian Amazon
Source: PLOS Glob Public Health. 2026 Nov 2;6(11):e0007553. doi: 10.1371/journal.pgph.0007553 (PMC13726989; doi:10.1371/journal.pgph.0007553)
Supplement: Supplementary file 12 [file pgph.0007553.s013.docx]

S10 Table. Distribution of spatial units across residual categories by geographic boundary, malaria species and model, with 95% Wilson score confidence intervals. Residuals are mean monthly observed minus predicted cases per 100 inhabitants. Overestimated combines the strong and moderate overestimation categories (residual below -0.01), underestimated combines the moderate and strong underestimation categories (residual above 0.01).

| Geographic boundary | Species | Model | n | Strong over n (%) | Moderate over n (%) | Calibrated n (%) | Moderate under n (%) | Strong under n (%) | Overestimated % (95% CI) | Underestimated % (95% CI) |
| --- | --- | --- | --- | --- | --- | --- | --- | --- | --- | --- |
| ANA | *P. falciparum* | ZINB | 33 | 17 (51.5) | 6 (18.2) | 5 (15.2) | 1 (3.0) | 4 (12.1) | 69.7 (52.7-82.6) | 15.2 (6.7-30.9) |
| ANA | *P. falciparum* | Random forest | 33 | 1 (3.0) | 11 (33.3) | 19 (57.6) | 0 (0.0) | 2 (6.1) | 36.4 (22.2-53.4) | 6.1 (1.7-19.6) |
| ANA | *P. falciparum* | Support vector machine | 33 | 1 (3.0) | 3 (9.1) | 20 (60.6) | 4 (12.1) | 5 (15.2) | 12.1 (4.8-27.3) | 27.3 (15.1-44.2) |
| ANA | *P. falciparum* | XGBoost | 33 | 0 (0.0) | 5 (15.2) | 26 (78.8) | 2 (6.1) | 0 (0.0) | 15.2 (6.7-30.9) | 6.1 (1.7-19.6) |
| ANA | *P. vivax* | ZINB | 33 | 16 (48.5) | 8 (24.2) | 4 (12.1) | 1 (3.0) | 4 (12.1) | 72.7 (55.8-84.9) | 15.2 (6.7-30.9) |
| ANA | *P. vivax* | Random forest | 33 | 6 (18.2) | 12 (36.4) | 9 (27.3) | 4 (12.1) | 2 (6.1) | 54.5 (38.0-70.2) | 18.2 (8.6-34.4) |
| ANA | *P. vivax* | Support vector machine | 33 | 4 (12.1) | 6 (18.2) | 12 (36.4) | 2 (6.1) | 9 (27.3) | 30.3 (17.4-47.3) | 33.3 (19.8-50.4) |
| ANA | *P. vivax* | XGBoost | 33 | 2 (6.1) | 11 (33.3) | 11 (33.3) | 6 (18.2) | 3 (9.1) | 39.4 (24.7-56.3) | 27.3 (15.1-44.2) |
| District | *P. falciparum* | ZINB | 53 | 7 (13.2) | 17 (32.1) | 18 (34.0) | 8 (15.1) | 3 (5.7) | 45.3 (32.7-58.5) | 20.8 (12.0-33.5) |
| District | *P. falciparum* | Random forest | 53 | 0 (0.0) | 10 (18.9) | 39 (73.6) | 3 (5.7) | 1 (1.9) | 18.9 (10.6-31.4) | 7.5 (3.0-17.9) |
| District | *P. falciparum* | Support vector machine | 53 | 0 (0.0) | 7 (13.2) | 32 (60.4) | 6 (11.3) | 8 (15.1) | 13.2 (6.5-24.8) | 26.4 (16.4-39.6) |
| District | *P. falciparum* | XGBoost | 53 | 0 (0.0) | 6 (11.3) | 41 (77.4) | 6 (11.3) | 0 (0.0) | 11.3 (5.3-22.6) | 11.3 (5.3-22.6) |
| District | *P. vivax* | ZINB | 53 | 20 (37.7) | 15 (28.3) | 2 (3.8) | 0 (0.0) | 16 (30.2) | 66.0 (52.6-77.3) | 30.2 (19.5-43.5) |
| District | *P. vivax* | Random forest | 53 | 0 (0.0) | 16 (30.2) | 30 (56.6) | 3 (5.7) | 4 (7.5) | 30.2 (19.5-43.5) | 13.2 (6.5-24.8) |
| District | *P. vivax* | Support vector machine | 53 | 5 (9.4) | 12 (22.6) | 19 (35.8) | 1 (1.9) | 16 (30.2) | 32.1 (21.1-45.5) | 32.1 (21.1-45.5) |
| District | *P. vivax* | XGBoost | 53 | 2 (3.8) | 12 (22.6) | 27 (50.9) | 6 (11.3) | 6 (11.3) | 26.4 (16.4-39.6) | 22.6 (13.5-35.5) |
| Hydrobasin level 6 | *P. falciparum* | ZINB | 13 | 3 (23.1) | 2 (15.4) | 4 (30.8) | 1 (7.7) | 3 (23.1) | 38.5 (17.7-64.5) | 30.8 (12.7-57.6) |
| Hydrobasin level 6 | *P. falciparum* | Random forest | 13 | 0 (0.0) | 3 (23.1) | 8 (61.5) | 0 (0.0) | 2 (15.4) | 23.1 (8.2-50.3) | 15.4 (4.3-42.2) |
| Hydrobasin level 6 | *P. falciparum* | Support vector machine | 13 | 1 (7.7) | 0 (0.0) | 6 (46.2) | 3 (23.1) | 3 (23.1) | 7.7 (1.4-33.3) | 46.2 (23.2-70.9) |
| Hydrobasin level 6 | *P. falciparum* | XGBoost | 13 | 0 (0.0) | 2 (15.4) | 9 (69.2) | 0 (0.0) | 2 (15.4) | 15.4 (4.3-42.2) | 15.4 (4.3-42.2) |
| Hydrobasin level 6 | *P. vivax* | ZINB | 13 | 6 (46.2) | 3 (23.1) | 0 (0.0) | 2 (15.4) | 2 (15.4) | 69.2 (42.4-87.3) | 30.8 (12.7-57.6) |
| Hydrobasin level 6 | *P. vivax* | Random forest | 13 | 2 (15.4) | 1 (7.7) | 7 (53.8) | 0 (0.0) | 3 (23.1) | 23.1 (8.2-50.3) | 23.1 (8.2-50.3) |
| Hydrobasin level 6 | *P. vivax* | Support vector machine | 13 | 1 (7.7) | 2 (15.4) | 3 (23.1) | 1 (7.7) | 6 (46.2) | 23.1 (8.2-50.3) | 53.8 (29.1-76.8) |
| Hydrobasin level 6 | *P. vivax* | XGBoost | 13 | 0 (0.0) | 4 (30.8) | 5 (38.5) | 1 (7.7) | 3 (23.1) | 30.8 (12.7-57.6) | 30.8 (12.7-57.6) |
| Hydrobasin level 7 | *P. falciparum* | ZINB | 76 | 45 (59.2) | 16 (21.1) | 10 (13.2) | 2 (2.6) | 3 (3.9) | 80.3 (70.0-87.7) | 6.6 (2.8-14.5) |
| Hydrobasin level 7 | *P. falciparum* | Random forest | 76 | 1 (1.3) | 21 (27.6) | 46 (60.5) | 6 (7.9) | 2 (2.6) | 28.9 (20.0-40.0) | 10.5 (5.4-19.4) |
| Hydrobasin level 7 | *P. falciparum* | Support vector machine | 76 | 8 (10.5) | 0 (0.0) | 45 (59.2) | 12 (15.8) | 11 (14.5) | 10.5 (5.4-19.4) | 30.3 (21.1-41.3) |
| Hydrobasin level 7 | *P. falciparum* | XGBoost | 76 | 1 (1.3) | 17 (22.4) | 48 (63.2) | 6 (7.9) | 4 (5.3) | 23.7 (15.5-34.4) | 13.2 (7.3-22.6) |
| Hydrobasin level 7 | *P. vivax* | ZINB | 76 | 35 (46.1) | 15 (19.7) | 6 (7.9) | 2 (2.6) | 18 (23.7) | 65.8 (54.6-75.5) | 26.3 (17.7-37.2) |
| Hydrobasin level 7 | *P. vivax* | Random forest | 76 | 6 (7.9) | 36 (47.4) | 17 (22.4) | 9 (11.8) | 8 (10.5) | 55.3 (44.1-65.9) | 22.4 (14.5-32.9) |
| Hydrobasin level 7 | *P. vivax* | Support vector machine | 76 | 4 (5.3) | 24 (31.6) | 20 (26.3) | 7 (9.2) | 21 (27.6) | 36.8 (26.9-48.1) | 36.8 (26.9-48.1) |
| Hydrobasin level 7 | *P. vivax* | XGBoost | 76 | 10 (13.2) | 25 (32.9) | 19 (25.0) | 12 (15.8) | 10 (13.2) | 46.1 (35.3-57.2) | 28.9 (20.0-40.0) |
