# Supplementary material for: Comparing watershed-based spatial units and administrative boundaries for malaria prediction in the Peruvian Amazon
Source: PLOS Glob Public Health. 2026 Nov 2;6(11):e0007553. doi: 10.1371/journal.pgph.0007553 (PMC13726989; doi:10.1371/journal.pgph.0007553)
Supplement: Supplementary file 13 [file pgph.0007553.s015.docx]

Rethinking Spatial Units for Malaria Prediction in Loreto, Peru: The Use of Watershed-Based Boundaries in the Amazon Rainforest

PGPH-D-25-02224 | PLOS Global Public Health

| Manuscript ID | PGPH-D-25-02224 |
| --- | --- |
| Journal | PLOS Global Public Health |
| Article title | Rethinking Spatial Units for Malaria Prediction in Loreto, Peru: The Use of Watershed-Based Boundaries in the Amazon Rainforest |
| Date | March 28, 2026 |

March 28, 2026

Helen Howard

Staff Editor

PLOS Global Public Health

**Re:** Revised submission PGPH-D-25-02224, “Rethinking Spatial Units for Malaria Prediction in Loreto, Peru: The Use of Watershed-Based Boundaries in the Amazon Rainforest”

Dear Ms. Howard,

On behalf of all authors, I would like to thank you and the reviewers for the careful and constructive evaluation of our manuscript. We found the comments highly valuable and have revised the paper extensively in response.

The revised manuscript has been strengthened in the areas emphasized in the decision letter. In particular, we clarified the prediction-focused study objective in the Introduction; corrected the statistical terminology so that the count model is consistently described as a zero-inflated negative binomial model; expanded the reporting of predictor selection and machine-learning hyperparameter optimization; revised the performance framework to better distinguish predictive accuracy from model parsimony; and added blocked spatiotemporal and temporal validation analyses to better assess generalizability.

We also strengthened the Discussion by situating our findings more explicitly within previous ecological malaria models from the Amazon region and by detailing how hydrobasin-based predictions could complement administrative malaria control in Loreto. In addition, we expanded the limitations to acknowledge more clearly that the models did not explicitly include spatial autocorrelation, seasonal autocorrelation, or lag structures, and that the surveillance data represent passive case detection.

Finally, we revised the title to specify Loreto, Peru, added a dedicated ethics statement clarifying that formal ethics approval was not required because the study used anonymized secondary aggregated surveillance data, and corrected the code repository link in the Data Availability statement.

We hope that the revised manuscript and enclosed point-by-point response adequately address the concerns raised and that the paper will now be suitable for further consideration in PLOS Global Public Health.

Sincerely,

Gabriel Carrasco-Escobar, MSc PhD

On behalf of all authors

Response to Reviewers

Below we respond point by point to each reviewer comment. Reviewer comments are reproduced in full, followed by our response and a brief indication of where the corresponding changes were made in the revised manuscript.

Reviewer 1

Comment 1

**Reviewer comment:** *The introduction should conclude with a clear statement that the study aims to compare predictive performance across boundary systems using out-of-sample metrics.*

**Response:** We thank the reviewer for this suggestion. We revised the final paragraph of the Introduction to state explicitly that the study compares predictive performance across administrative and watershed-based boundary systems using out-of-sample evaluation metrics. This clarification makes the prediction-focused objective of the paper more explicit and better aligns the framing of the study with the evaluation strategy used in the revised manuscript.

**Changes in manuscript:** Introduction, final paragraph.

Comment 2

**Reviewer comment:** *The model specification as “zero-inflated Poisson regression with a negative binomial family” is non-standard and should be clarified as either a ZINB or a ZIP with overdispersion correction.*

**Response:** We appreciate this important observation. We corrected the terminology throughout the manuscript and now consistently describe the statistical count model as a zero-inflated negative binomial (ZINB) model. The previous phrasing was imprecise, and the revised text now clearly reflects the model actually fitted to the malaria count data.

**Changes in manuscript:** Abstract; Methods Section 2.4.2 (Modeling); figure/table terminology where applicable.

Comment 3

**Reviewer comment:** *Statistical assessment of zero inflation should be included, for example through performance comparison between models with and without zero inflation. Update the abstract based on the response to this comment.*

**Response:** Thank you for this suggestion. We added text to the Methods clarifying that alternative specifications without a zero-inflation component were evaluated during model development, but these models showed poorer fit and convergence problems in the presence of a high proportion of zero counts. On this basis, the zero-inflated specification was retained. We also revised the Abstract so that it now explicitly reports the use of ZINB regression rather than the earlier ambiguous terminology.

**Changes in manuscript:** Abstract; Methods Section 2.4.2 (Modeling).

Comment 4

**Reviewer comment:** *The manuscript should explicitly state whether spatial and/or temporal autocorrelation were incorporated in the models. Without this information, it is difficult to evaluate whether the cross-validation strategy appropriately accounted for data dependencies.*

**Response:** We agree that this point required clearer reporting. The revised manuscript now states explicitly that spatial and temporal autocorrelation were not directly parameterized within the fitted models using explicit correlation structures or lag terms. To reduce overly optimistic performance estimates arising from dependence in the data, we added a blocked spatiotemporal cross-validation framework that separates both future time periods and held-out spatial units during evaluation. We also acknowledge in the limitations that this validation strategy does not replace explicit modeling of autocorrelation.

**Changes in manuscript:** Methods Section 2.4.5 (Spatiotemporal cross-validation); Discussion, limitations paragraph.

Comment 5

**Reviewer comment:** *For the machine-learning algorithms, the parameter ranges explored should be reported in full, including for RF, SVM, and XGBoost.*

**Response:** We agree and have expanded the Methods accordingly. A dedicated subsection now reports the hyperparameter optimization strategy and the parameter ranges explored for Random Forest, SVM with radial kernel, and XGBoost. We also provide the optimal configurations identified for each disease and spatial unit in a supplementary table to improve transparency and reproducibility.

**Changes in manuscript:** Methods Section 2.4.3 (Hyperparameter optimisation); Supplementary Table 3.

Comment 6

**Reviewer comment:** *The current set of performance metrics is limited and partially redundant (RMSE and RSE). Additional measures such as information criteria and cross-validation error metrics should be included.*

**Response:** We appreciate this observation and revised the performance framework. Residual Standard Error (RSE) has been removed. The manuscript now distinguishes predictive accuracy from model parsimony by reporting RMSE together with Akaike Information Criterion (AIC) for the statistical models. In addition, the revised manuscript includes blocked spatiotemporal and temporal validation analyses to provide a more rigorous assessment of generalizability beyond the original model fitting strategy.

**Changes in manuscript:** Methods Sections 2.4.4 and 2.4.5; Results Section 3.4; Table 2; Supplementary Table 5.

Comment 7

**Reviewer comment:** *The discussion should be strengthened by providing more detailed comparison with ecological models previously developed for the Amazon region.*

**Response:** Thank you for this suggestion. We expanded the Discussion to better situate our findings within the Amazon malaria literature, including prior work on hydrology, wetlands, vector ecology, riverine transmission, and connectivity in the Peruvian Amazon and related settings. This revision helps clarify that the contribution of our study is not only predictive comparison across algorithms, but also the demonstration that ecologically meaningful boundary systems may improve climate-malaria modeling in Amazonian environments.

**Changes in manuscript:** Discussion, first half of the section.

Comment 8

**Reviewer comment:** *There should be stronger acknowledgement in the limitations that spatial autocorrelation was not incorporated. Furthermore, no seasonal autocorrelation or lag terms appear to have been considered. The limitations of relying on passive surveillance data should also be explicitly stated.*

**Response:** We agree and strengthened the limitations section accordingly. The revised manuscript now states explicitly that the models did not include direct spatial autocorrelation terms, seasonal autocorrelation structures, or lagged effects. We also expanded the discussion of surveillance limitations by clarifying that the analysis relies on passive case detection, which may undercapture asymptomatic, submicroscopic, or non-care-seeking infections and therefore primarily reflects reported clinical malaria cases.

**Changes in manuscript:** Discussion, limitations paragraph.

Comment 9

**Reviewer comment:** *The link to the code repository does not work and should be provided to ensure reproducibility. The repository should include the full codebase together with session information to allow robust critical appraisal.*

**Response:** We thank the reviewer for noting this issue. The repository link in the Data Availability statement has been corrected and verified so that readers can access the study repository directly. We also revised the reproducibility statement to make the availability of the analytical code explicit. This change was made to facilitate critical appraisal and reuse of the analytical workflow.

**Changes in manuscript:** Data Availability statement.

Comment 10

**Reviewer comment:** *As this is a prediction study, the manuscript should be aligned with the EPIFORGE guidelines (2020) for reporting epidemiological prediction studies.*

**Response:** We agree. The revised manuscript now states explicitly that the study was conducted and reported in accordance with EPIFORGE 2020. In practical terms, we strengthened the reporting of the study objective, model development, validation strategy, and performance assessment so that the manuscript better reflects reporting expectations for epidemiological prediction studies.

**Changes in manuscript:** Methods Section 2.1 (Study design) and related reporting revisions throughout the manuscript.

Comment 11

**Reviewer comment:** *The title should include that the study was carried out in Loreto, Peru.*

**Response:** We agree and revised the title accordingly so that the geographic setting is explicit from the outset.

**Changes in manuscript:** Title revised to: “Rethinking Spatial Units for Malaria Prediction in Loreto, Peru: The Use of Watershed-Based Boundaries in the Amazon Rainforest.”

Comment 12

**Reviewer comment:** *Line 219: the term “optimised set” is vague. The variables that passed the selection phase should be listed explicitly, not only referred to in Supplementary Table 2.*

**Response:** Thank you for this suggestion. We revised the Methods text to clarify how the final predictor sets were defined after backward selection and Elastic Net regularization. Because the study includes multiple boundary-specific and species-specific predictor sets, we retained a concise description in the main text but now point readers directly to Supplementary Table 2, where the selected variables are listed explicitly for each set.

**Changes in manuscript:** Methods Section 2.4.1 (Predictors selection); Supplementary Table 2.

Comment 13

**Reviewer comment:** *The manuscript should note that no ethics approval was required for this study.*

**Response:** We agree. We added a dedicated ethics statement clarifying that the study used anonymized secondary surveillance data analyzed at aggregated spatial levels and therefore did not require formal ethics committee approval under the applicable institutional and national guidance.

**Changes in manuscript:** Methods Section 2.4.6 (Ethics).

Reviewer 2

Comment 1

**Reviewer comment:** *The authors have geocoded village malaria data. Instead of only comparing methods that rely on aggregations to districts or hydrobasins, they could have extracted proximity to hydrobasins (by type) by village and do analyses that compare to the aggregated approaches - where village location vs proximity to hydrobasin by type is compared to district boundary vs hydrobasin polygon.*

**Response:** We appreciate this thoughtful suggestion. A village-level proximity analysis would indeed address an important related question. However, the objective of the present study was narrower and operationally oriented: to compare alternative areal frameworks for malaria prediction under conditions similar to those faced by surveillance and control programs, where exposures are summarized over polygons and decisions are typically implemented using area-based units. A point-level proximity design would require a different analytical framework and would answer a complementary, but distinct, question. To keep the study focused, we retained the polygon-based comparison and clarified its operational rationale in the revised manuscript.

**Changes in manuscript:** Discussion, policy and interpretation framing; scope clarified in the revised text.

Comment 2

**Reviewer comment:** *My suggestion for above analysis is related to moving the study from comparing polygon to polygon comparison to also addressing the important concerns around the ‘spatial support problem’ in geospatial analysis. Spatial support is the size, shape, and orientation of the area or volume where a measurement is made. The variability of the variables is affected by these differences, and it will be interesting to see how the scaling effects inform the results when village analysis is compared to polygon level analysis. I think this will offer greater insights into the relationship between malaria cases, population locations and hydrobasins that are relevant to policy.*

**Response:** Thank you for highlighting this conceptual issue. We agree that spatial support is highly relevant here. In the revised Discussion, we now make this point more explicit and note that the comparison across districts, Hydrobasin level 6, Hydrobasin level 7, and ANA boundaries can be interpreted as an applied assessment of how alternative spatial supports influence predictor variability and model performance. At the same time, we acknowledge that a full village-to-polygon multiscale analysis would be a valuable next step for future work.

**Changes in manuscript:** Discussion, expanded conceptual interpretation of boundary choice.

Comment 3

**Reviewer comment:** *The authors aggregate malaria data from a 10-year period. While the limitations of these aggregations are acknowledged, I think there is a way of still comparing time periods that would add to the analysis. For example, authors could compare the aggregated 10-year data to the first five years and the second five years. This comparison would help describe if changes over time affect the relationships that the authors observe.*

**Response:** We appreciate this suggestion and incorporated an additional temporal assessment in response. The revised manuscript now includes a blocked spatiotemporal validation framework together with a temporal holdout analysis using 2009-2014 for training and 2015-2018 for testing. These results are presented in the Results and supplementary material and allow us to evaluate whether the relative performance patterns remain consistent when the models are assessed on future periods. Broadly, the revised analyses support the same overall conclusion, with Hydrobasin level 7 continuing to show the most favorable performance across species.

**Changes in manuscript:** Methods Section 2.4.5; Results Section 3.4; Figure 5; Supplementary Table 5.

Comment 4

**Reviewer comment:** *Given policies are designed for and implemented in administrative units, what are the policy actions that emerge from their analyses that can be used to tailor the malaria response in Loreto?*

**Response:** We agree that this point needed to be made more concrete. We expanded the Discussion to explain how hydrobasin-based predictions could be used as a complementary layer of risk stratification nested within existing administrative decision-making. In practice, this can help identify high-risk hydrological subregions within districts to prioritize vector control, focal larval source management, targeted indoor residual spraying, active surveillance, and more efficient allocation of diagnostics and response resources along river-connected transmission corridors. We believe this clarification strengthens the translational relevance of the study for malaria control in Loreto.

**Changes in manuscript:** Discussion, final policy-oriented paragraph.
