# Supplementary material for: Comparing watershed-based spatial units and administrative boundaries for malaria prediction in the Peruvian Amazon
Source: PLOS Glob Public Health. 2026 Nov 2;6(11):e0007553. doi: 10.1371/journal.pgph.0007553 (PMC13726989; doi:10.1371/journal.pgph.0007553)
Supplement: Peer review history

# Peer review: Decision Letter 0

**DOI:** 10.1371/journal.pgph.0007553.r001

**Authors:** Helen Howard

17 Dec 2025

PGPH-D-25-02224

Rethinking Spatial Units for Malaria Prediction: The Use of Watershed-Based Boundaries in the Amazon Rainforest

PLOS Global Public Health

Dear Dr. Ascencio,

Thank you for submitting your manuscript to PLOS Global Public Health. After careful consideration, we feel that it has merit but does not fully meet PLOS Global Public Health’s publication criteria as it currently stands. Therefore, we invite you to submit a revised version of the manuscript that addresses the points raised during the review process.

The manuscript has been evaluated by two reviewers, and their comments are available below.

The reviewers have raised a number of concerns that need attention. In particular, they request additional information on methodological and modelling aspects of the study, and improvements to the Discussion.

Could you please revise the manuscript to carefully address the concerns raised?

Please submit your revised manuscript by Jan 31 2026 11:59PM. If you will need more time than this to complete your revisions, please reply to this message or contact the journal office at globalpubhealth@plos.org. When you're ready to submit your revision, log on to https://www.editorialmanager.com/pgph/ and select the 'Submissions Needing Revision' folder to locate your manuscript file.

Please include the following items when submitting your revised manuscript:

- A letter that responds to each point raised by the editor and reviewer(s). You should upload this letter as a separate file labeled 'Response to Reviewers'.
- A marked-up copy of your manuscript that highlights changes made to the original version. You should upload this as a separate file labeled 'Revised Manuscript with Track Changes'.
- An unmarked version of your revised paper without tracked changes. You should upload this as a separate file labeled 'Manuscript'.

Guidelines for resubmitting your figure files are available below the reviewer comments at the end of this letter.

We look forward to receiving your revised manuscript.

Kind regards,

Helen Howard

Staff Editor

PLOS Global Public Health

Journal Requirements:

If the reviewer comments include a recommendation to cite specific previously published works, please review and evaluate these publications to determine whether they are relevant and should be cited. There is no requirement to cite these works unless the editor has indicated otherwise.

Additional Editor Comments (if provided):

[Note: HTML markup is below. Please do not edit.]

Reviewers' comments:

Reviewer's Responses to Questions

**Comments to the Author**

1. Does this manuscript meet PLOS Global Public Health’s publication criteria?

Reviewer #1: Yes

Reviewer #2: Yes

**********

2. Has the statistical analysis been performed appropriately and rigorously?

Reviewer #1: I don't know

Reviewer #2: Yes

**********

3. Have the authors made all data underlying the findings in their manuscript fully available (please refer to the Data Availability Statement at the start of the manuscript PDF file)?

The PLOS Data policy

Reviewer #1: No

Reviewer #2: No

**********

4. Is the manuscript presented in an intelligible fashion and written in standard English?

Reviewer #1: Yes

Reviewer #2: Yes

**********

Reviewer #1: Dear authors,

Thank you for sharing this work with the community. It is an interesting modelling exercise that raises the important question of whether modelling efforts should be conducted solely at administrative-unit level, constrained by the limitations of surveillance systems. The manuscript, however, requires substantial modification to improve robustness, interpretability, and reproducibility.

I provide my observations below.

Kind regards

---

Major comments

- The introduction should conclude with a clear statement that the study aims to compare predictive performance across boundary systems using out-of-sample metrics.

- The model specification as “zero-inflated Poisson regression with a negative binomial family” is non-standard and should be clarified as either a ZINB or a ZIP with overdispersion correction.

- Statistical assessment of zero inflation should be included, for example through performance comparison between models with and without zero inflation. Update the abstract based on the response to this comment.

- The manuscript should explicitly state whether spatial and/or temporal autocorrelation were incorporated in the models. Without this information, it is difficult to evaluate whether the cross-validation strategy appropriately accounted for data dependencies.

- For the machine-learning algorithms, the parameter ranges explored should be reported in full, including for RF, SVM, and XGBoost.

- The current set of performance metrics is limited and partially redundant (RMSE and RSE). Additional measures such as information criteria and cross-validation error metrics should be included.

- The discussion should be strengthened by providing more detailed comparison with ecological models previously developed for the Amazon region.

- There should be stronger acknowledgement in the limitations that spatial autocorrelation was not incorporated. Furthermore, no seasonal autocorrelation or lag terms appear to have been considered. The limitations of relying on passive surveillance data should also be explicitly stated.

- The link to the code repository does not work and should be provided to ensure reproducibility. The repository should include the full codebase together with session information to allow robust critical appraisal.

- As this is a prediction study, the manuscript should be aligned with the EPIFORGE guidelines (2020) for reporting epidemiological prediction studies.

Minor comments

- The title should include that the study was carried out in Loreto, Peru.

- Line 219: the term “optimised set” is vague. The variables that passed the selection phase should be listed explicitly, not only referred to in Supplementary Table 2.

- The manuscript should note that no ethics approval was required for this study.

Reviewer #2: General comments

The paper is well written, easy to understand with replicable approaches that relies on robust statistical and machine learning methods. The authors address the accuracy of predictions of malaria cases comparing administrative boundaries to ecological aggregations, using hydrobasins, in Loreto region of Peru. Some important limitations are acknowledged.

Major comments

My main concerns are related to the study conceptualization given the available data and their policy implications.

1. The authors have geocoded village malaria data. Instead of only comparing methods that rely on aggregations to districts or hydrobasins, they could have extracted proximity to hydrobasins (by type) by village and do analyses that compare to the aggregated approaches - where village location vs proximity to hydrobasin by type is compared to district boundary vs hydrobasin polygon.

2. My suggestion for above analysis is related to moving the study from comparing polygon to polygon comparison to also addressing the important concerns around the ‘spatial support problem’ in geospatial analysis. Spatial support is the size, shape, and orientation of the area or volume where a measurement is made. The variability of the variables is affected by these differences, and it will be interesting to see how the scaling effects inform the results when village analysis is compared to polygon level analysis. I think this will offer greater insights into the relationship between malaria cases, population locations and hydrobasins that are relevant to policy.

3. The authors aggregate malaria data from a 10-year period. While the limitations of these aggregations are acknowledged, I think there is a way of still comparing time periods that would add to the analysis. For example, authors could compare the aggregated 10-year data to the first five years and the second five years. This comparison would help describe if changes over time affect the relationships that the authors observe.

4. Given policies are designed for and implemented in administrative units, what are the policy actions that emerge from their analyses that can be used to tailor the malaria response in Loreto?

**********

what does this mean?). If published, this will include your full peer review and any attached files.

**Do you want your identity to be public for this peer review?**  If you choose “no”, your identity will remain anonymous but your review may still be made public.

For information about this choice, including consent withdrawal, please see our Privacy Policy

Reviewer #1: No

Reviewer #2: No

**********

[NOTE: If reviewer comments were submitted as an attachment file, they will be attached to this email and accessible via the submission site. Please log into your account, locate the manuscript record, and check for the action link "View Attachments". If this link does not appear, there are no attachment files.]

# Author comment: Author response to Decision Letter 1

**DOI:** 10.1371/journal.pgph.0007553.r002

29 Mar 2026

Attachment Submitted filename: PGPH_D_25_02224_Response_to_Reviewers.docx

# Peer review: Decision Letter 1

**DOI:** 10.1371/journal.pgph.0007553.r003

**Authors:** Miquel Vall-llosera Camps

14 Jul 2026

PGPH-D-25-02224R1

Rethinking Spatial Units for Malaria Prediction in Loreto, Peru: The Use of Watershed-Based Boundaries in the Amazon Rainforest

PLOS Global Public Health

Dear Dr. Ascencio,

Thank you for submitting your manuscript to PLOS Global Public Health. After careful consideration, we feel that it has merit but does not fully meet PLOS Global Public Health’s publication criteria as it currently stands. Therefore, we invite you to submit a revised version of the manuscript that addresses the points raised during the review process.

Please submit your revised manuscript by Sep 11 2026 11:59PM. If you will need more time than this to complete your revisions, please reply to this message or contact the journal office at globalpubhealth@plos.org. When you're ready to submit your revision, log on to https://www.editorialmanager.com/pgph/ and select the 'Submissions Needing Revision' folder to locate your manuscript file.

Please include the following items when submitting your revised manuscript:

- A letter that responds to each point raised by the editor and reviewer(s). You should upload this letter as a separate file labeled 'Response to Reviewers'.
- A marked-up copy of your manuscript that highlights changes made to the original version. You should upload this as a separate file labeled 'Revised Manuscript with Track Changes'.
- An unmarked version of your revised paper without tracked changes. You should upload this as a separate file labeled 'Manuscript'.

Guidelines for resubmitting your figure files are available below the reviewer comments at the end of this letter.

As the corresponding author, your ORCID iD is verified in the submission system and will appear in the published article. PLOS supports the use of ORCID, and we encourage all coauthors to register for an ORCID iD and use it as well. Please encourage your coauthors to verify their ORCID iD within the submission system before final acceptance, as unverified ORCID iDs will not appear in the published article. *Only*  the individual author can complete the verification step; PLOS staff *cannot*  verify ORCID iDs on behalf of authors.

We look forward to receiving your revised manuscript.

Kind regards,

Miquel Vall-llosera Camps

Staff Editor

PLOS Global Public Health

Journal Requirements:

If the reviewer comments include a recommendation to cite specific previously published works, please review and evaluate these publications to determine whether they are relevant and should be cited. There is no requirement to cite these works unless the editor has indicated otherwise.

Additional Editor Comments:

It was considered necessary to invite additional reviewers after the previous reviewers became unavailable.

[Note: HTML markup is below. Please do not edit.]

Reviewers' comments:

Reviewer's Responses to Questions

**Comments to the Author**

Reviewer #3: (No Response)

Reviewer #4: (No Response)

Reviewer #5: (No Response)

**********

publication criteria?

Reviewer #3: No

Reviewer #4: Yes

Reviewer #5: Partly

**********

3. Has the statistical analysis been performed appropriately and rigorously?

Reviewer #3: N/A

Reviewer #4: Yes

Reviewer #5: No

**********

4. Have the authors made all data underlying the findings in their manuscript fully available (please refer to the Data Availability Statement at the start of the manuscript PDF file)?

The PLOS Data policy

Reviewer #3: Yes

Reviewer #4: Yes

Reviewer #5: Yes

**********

5. Is the manuscript presented in an intelligible fashion and written in standard English?

Reviewer #3: Yes

Reviewer #4: (No Response)

Reviewer #5: Yes

**********

Reviewer #3: 1. Authors need to address the MAUP/scale confound directly. Add an explicit discussion of the Modifiable Areal Unit Problem, and ideally a supplementary analysis demonstrating that hydrobasin level 7's RMSE advantage is not simply attributable to it having the smallest average case counts of any boundary type (Table 1).

2. Revise the cross-boundary AIC comparisons and the cross-application of boundary-specific models to other boundaries' covariate structures (Section 2.4.2/3.2).

3. Reconcile the 88%/12% (cited national figure, ref. 5) vs. 79.48%/20.52% (this study's data) P. vivax/P. falciparum split — a brief explanatory sentence is needed.

4. Correct the abstract's percentage-reduction sentences so raw RMSE values and percentage reductions are not conflated in the same clause.

5. Provide the missing total count of ANA hydrobasin units, and consider adding a summary table comparing the four boundary types by number of units, mean unit area, and mean population, to let readers directly assess whether finer resolution alone (rather than hydrological "ecological relevance") explains the result pattern.

6. Fix the typo errors. (example : "Lotero" → "Loreto" (line 141), "Hydrostatic Release Units" → "Hydrologic Response Units" (lines 502–503), consistent with your own ref. 27.)

7. Fix reference 1's URL (currently points to the 2021, not 2023, report page).

8. Verify in-text citation of references 47 and 48.

9.

Reviewer #4: I have two minor comments:

1. Clarify the population offset: The Methods neverstate how population was derived. Please specify the source, the allocationmethod (particularly for the hydrobasins), and whether population varied byyear (also for the districts).

2. Sample size discrepancy: Hydrobasin level 7 isreported as 78 units  in the text, 76(Figure 1), and 53 (Figure 4d).

Reviewer #5: TITLE

The title contains unnecessary words and, in some cases, unscientific words. I propose that the authors consider revising the title to: “Comparing Watershed-Based Spatial Units and Administrative Boundaries for Malaria Prediction in the Peruvian Amazon”.

INTRODUCTION

The exact gap is unclear. Stating that watershed-based boundaries are scarce does not make it a gap. Use proper literature synthesis to demonstrate why administrative units produce inferior epidemiological inference by discussing fundamental concepts such as the Modifiable Areal Unit Problem (MAUP), ecological misalignment, spatial aggregation bias, and political versus ecological boundaries. Support your review of existing works with epidemiological theory that discusses ecological fallacy, modifiable areal unit problem (MAUP), and spatial misalignment. Include more forecasting-based literature to support your work. Strengthen the knowledge gap by discussing whether there is evidence in current literature that supports your initial position that current works implicitly assume that administrative units appropriately represent environmentally driven transmission processes, yet this assumption has rarely been empirically evaluated. You may review literature around the interaction of meteorological controls, hydrological controls, and vegetation controls in shaping vector ecology. Briefly summarise previous forecasting frameworks before introducing the spatial-unit problem. Reduce excessive use of statistics in the introduction section and provide a compelling storyline.

METHODS

In your design, explicitly provide clarity by distinguishing between which of the models are for assessing explanatory relationships and which are for predictive modelling. Remove redundancies from the study area write-up. Remove all text that has no methodological relevance to the study objective. For example, do away with information about historical descriptions of the creation of districts and provinces. Provide a concise justification for selecting your three specific scales, such as Hydrobasin level 6, Hydrobasin level 7, and ANA. Explain the rationale behind using different predictor-selection approaches for different modelling families. Why did you use Elastic Net and PCA only for specific models? Provide a metric-based justification for assessing model fit; otherwise, it remains subjective. Shed more light on the Cross-boundary model transfer concept. For ML models, report additional metrics such as MAE, R², calibration, and prediction intervals.

RESULTS

Support your reported statistics with interpretation. Avoid repetition. The result section should report on the residual analysis, explaining specific regions that show systematic over- or underestimation. Quantify clearly the metric difference for comparable validation settings. Identify the most influential predictors. Compute and report 95% confidence intervals for each reported estimate. Provide evidence of statistical comparison by reporting Diebold–Mariano tests or bootstrap comparisons.

DISCUSSION

The authors should directly and explicitly state that their contribution is challenging the assumption that administrative boundaries are the correct spatial unit for infectious disease forecasting. Authors should be able to understand at this point whether their findings state whether their results confirm, contradict, or extend previous prediction research. Discuss MAUP and explain that from your results, different spatial aggregation units alter statistical relationships, and Hydrobasin Level 7 reduces aggregation bias by better aligning with environmental transmission processes. Discuss the observed predictive performance consistency in your Algorithm and geographical location. Avoid speculative inferences.

**********

what does this mean?). If published, this will include your full peer review and any attached files.

**Do you want your identity to be public for this peer review?**  If you choose “no”, your identity will remain anonymous but your review may still be made public.

For information about this choice, including consent withdrawal, please see our Privacy Policy

Reviewer #3: No

Reviewer #4: **Yes:**  Adrienne Epstein

Reviewer #5: No

**********

[NOTE: If reviewer comments were submitted as an attachment file, they will be attached to this email and accessible via the submission site. Please log into your account, locate the manuscript record, and check for the action link "View Attachments". If this link does not appear, there are no attachment files.]

# Author comment: Author response to Decision Letter 2

**DOI:** 10.1371/journal.pgph.0007553.r004

11 Sep 2026

Attachment Submitted filename: rebuttal_letter_hydrobasins_GabrielCarrasco.docx.pdf

# Peer review: Decision Letter 2

**DOI:** 10.1371/journal.pgph.0007553.r005

**Authors:** Xin Sun

24 Sep 2026

PGPH-D-25-02224R2

Comparing watershed-based spatial units and administrative boundaries for malaria prediction in the Peruvian Amazon

PLOS Global Public Health

Dear Dr. Ascencio,

Thank you for submitting your manuscript to PLOS Global Public Health. After careful consideration, we feel that it has merit but does not fully meet PLOS Global Public Health’s publication criteria as it currently stands. Therefore, we invite you to submit a revised version of the manuscript that addresses the points raised during the review process.

A previous reviewer has evaluated your work and raised further concerns regarding the content of the manuscript. Please read carefully and address them in your revision.

Please submit your revised manuscript by Nov 23 2026 11:59PM. If you will need more time than this to complete your revisions, please reply to this message or contact the journal office at globalpubhealth@plos.org. When you're ready to submit your revision, log on to https://www.editorialmanager.com/pgph/ and select the 'Submissions Needing Revision' folder to locate your manuscript file.

Please include the following items when submitting your revised manuscript:

- A letter that responds to each point raised by the editor and reviewer(s). You should upload this letter as a separate file labeled 'Response to Reviewers'.
- A marked-up copy of your manuscript that highlights changes made to the original version. You should upload this as a separate file labeled 'Revised Manuscript with Track Changes'.
- An unmarked version of your revised paper without tracked changes. You should upload this as a separate file labeled 'Manuscript'.

Guidelines for resubmitting your figure files are available below the reviewer comments at the end of this letter.

As the corresponding author, your ORCID iD is verified in the submission system and will appear in the published article. PLOS supports the use of ORCID, and we encourage all coauthors to register for an ORCID iD and use it as well. Please encourage your coauthors to verify their ORCID iD within the submission system before final acceptance, as unverified ORCID iDs will not appear in the published article. *Only*  the individual author can complete the verification step; PLOS staff *cannot*  verify ORCID iDs on behalf of authors.

We look forward to receiving your revised manuscript.

Kind regards,

Xin Sun, PhD

Staff Editor

PLOS Global Public Health

Journal Requirements:

If the reviewer comments include a recommendation to cite specific previously published works, please review and evaluate these publications to determine whether they are relevant and should be cited. There is no requirement to cite these works unless the editor has indicated otherwise.

Additional Editor Comments (if provided):

[Note: HTML markup is below. Please do not edit.]

Reviewers' comments:

Reviewer's Responses to Questions

**Comments to the Author**

Reviewer #5: (No Response)

**********

publication criteria?

Reviewer #5: Partly

**********

3. Has the statistical analysis been performed appropriately and rigorously?

Reviewer #5: No

**********

4. Have the authors made all data underlying the findings in their manuscript fully available (please refer to the Data Availability Statement at the start of the manuscript PDF file)?

The PLOS Data policy

Reviewer #5: Yes

**********

5. Is the manuscript presented in an intelligible fashion and written in standard English?

Reviewer #5: Yes

**********

Reviewer #5: The manuscript has improved following the previous review. However, some issues still need to be resolved.

The authors should strengthen the introduction by providing an epidemiological bridge between spatial-statistical theory and malaria transmission ecology.

Strengthen the Methods section to indicate whether the choice of HydroBASINS Levels 6 and 7 was based on an a priori criterion or an empirical assessment of alternative spatial resolutions. Remove descriptive material concerning territorial hierarchy, governance functions, and institutional organization that does not contribute directly to the analytical design. Retain the information required to define the administrative spatial unit used for aggregation and comparison.

The justification for zero inflation should also be supported by explicit comparative diagnostics rather than the proportion of observed zeros alone. Clarify whether ZINB is being used for inferential estimation of environmental associations, predictive benchmarking, or both. Please make these analytical roles explicit and maintain a non-causal interpretation of regression coefficients. Please report the hyperparameters and tuning ranges for RF, SVM, and XGBoost.

Explain how monthly loss differentials were constructed for Diebold–Mariano tests when the competing boundary systems contain different numbers and configurations of spatial units, and justify the four-month Newey–West lag. Confidence intervals around RMSE quantify uncertainty in a performance statistic and should not be interpreted as prediction intervals for individual malaria outcomes. Please report or justify the omission of these requested predictive assessments.

**********

what does this mean?). If published, this will include your full peer review and any attached files.

**Do you want your identity to be public for this peer review?**  If you choose “no”, your identity will remain anonymous but your review may still be made public.

For information about this choice, including consent withdrawal, please see our Privacy Policy

Reviewer #5: No

**********

[NOTE: If reviewer comments were submitted as an attachment file, they will be attached to this email and accessible via the submission site. Please log into your account, locate the manuscript record, and check for the action link "View Attachments". If this link does not appear, there are no attachment files.]

# Author comment: Author response to Decision Letter 3

**DOI:** 10.1371/journal.pgph.0007553.r006

1 Oct 2026

Attachment Submitted filename: Response_to_Reviewers_R2resp.pdf

# Peer review: Decision Letter 3

**DOI:** 10.1371/journal.pgph.0007553.r007

**Authors:** Sheetal Silal

13 Oct 2026

Comparing watershed-based spatial units and administrative boundaries for malaria prediction in the Peruvian Amazon

PGPH-D-25-02224R3

Dear Ascencio,

We are pleased to inform you that your manuscript 'Comparing watershed-based spatial units and administrative boundaries for malaria prediction in the Peruvian Amazon' has been provisionally accepted for publication in PLOS Global Public Health.

Before your manuscript can be formally accepted you will need to complete some formatting changes, which you will receive in a follow up email. A member of our team will be in touch with a set of requests.

Please note that your manuscript will not be scheduled for publication until you have made the required changes, so a swift response is appreciated.

IMPORTANT: The editorial review process is now complete. PLOS will only permit corrections to spelling, formatting or significant scientific errors from this point onwards. Requests for major changes, or any which affect the scientific understanding of your work, will cause delays to the publication date of your manuscript.

If your institution or institutions have a press office, please notify them about your upcoming paper to help maximize its impact. If they'll be preparing press materials, please inform our press team as soon as possible -- no later than 48 hours after receiving the formal acceptance. Your manuscript will remain under strict press embargo until 2 pm Eastern Time on the date of publication. For more information, please contact globalpubhealth@plos.org.

Thank you again for supporting Open Access publishing; we are looking forward to publishing your work in PLOS Global Public Health.

Best regards,

Sheetal Prakash Silal, Ph.D.

Academic Editor

PLOS Global Public Health

***********************************************************

Reviewer Comments (if any, and for reference):

Reviewer's Responses to Questions

**Comments to the Author**

Reviewer #5: All comments have been addressed

**********

publication criteria?

Reviewer #5: Yes

**********

3. Has the statistical analysis been performed appropriately and rigorously?

Reviewer #5: Yes

**********

4. Have the authors made all data underlying the findings in their manuscript fully available (please refer to the Data Availability Statement at the start of the manuscript PDF file)?

The PLOS Data policy

Reviewer #5: Yes

**********

5. Is the manuscript presented in an intelligible fashion and written in standard English?

Reviewer #5: Yes

**********

Reviewer #5: I have no further issues.

**********

what does this mean?). If published, this will include your full peer review and any attached files.

**Do you want your identity to be public for this peer review?**  If you choose “no”, your identity will remain anonymous but your review may still be made public.

For information about this choice, including consent withdrawal, please see our Privacy Policy

Reviewer #5: No

**********
